# Application of Artificial Intelligence in Predicting Coal Mine Disaster Risks: A Review

**DOI:** 10.3390/s25216586

**Published:** 2025-10-26

**Authors:** Peiyan Lu, Yingjie Liu, Yuntao Liang, Dawei Cui

**Affiliations:** 1 China Coal Research Institute, Beijing 100013, China; 13920348191@163.com (P.L.); cuidawei@mail.ccri.ccteg.cn (D.C.); 2 Beijing Technology Research Branch, Tiandi Science & Technology Co., Ltd., Beijing 100013, China

**Keywords:** artificial intelligence, risk prediction, machine learning, coal mine disaster, forecasting model

## Abstract

The production environments of coal mines are inherently complex, with interrelated disaster risks that challenge safety management. Current prediction systems struggle with fragmented data, limited mechanistic understanding, and inadequate early warnings, falling short of modern coal mine safety needs. This paper advances the thesis that artificial intelligence, including machine learning, deep learning, and Large Language Model, provides essential tools for overcoming these prediction challenges in coal mining. We review AI-based approaches for forecasting coal and gas outbursts, mine fires, water disasters, roof collapses, and dust disasters, analyzing them through technical principles, application scenarios, and empirical outcomes. The analysis clarifies how AI improves risk prediction accuracy, enhances data integration, and enables smarter decision-making for safety. By examining the five major hazards, we highlight ongoing challenges in AI implementation and outline pathways for future development, emphasizing the importance of large models and autonomous agents. Our findings support the creation of advanced AI-driven safety and early warning systems for coal mines.

## 1. Introduction

With the rapid advancement of industrialization and informatization, coal mine disaster monitoring has become a crucial component in ensuring mining safety. The major types of coal mine disasters include gas disaster, mine fires, mine water disaster, roof disaster, and coal dust disaster. These hazards not only lead to casualties but also affect enterprise efficiency and energy security (Challenge and path of high-quality development of coal mine intelligent construction [1]. China is a major coal-producing nation globally. Due to its complex geological conditions and high disaster risks, it has become one of the countries with the most concentrated research in coal mine disaster prediction. Therefore, this paper focuses on China as its research subject. Unless otherwise specified, terms such as “nationwide,” “our country,” and “domestic” refer to China. According to statistics, a total of 168 coal mine accidents occurred nationwide in 2022, resulting in 245 fatalities, representing year-on-year increases of 85% and 38% [2]. In recent years, artificial intelligence technologies, especially in image recognition and deep learning, have achieved significant breakthroughs and are increasingly applied to coal mine safety. These applications have markedly improved the accuracy and timeliness of disaster monitoring, and related research has demonstrated promising progress both domestically and internationally [3,4,5,6].

Although artificial intelligence has made significant progress in coal mine disaster prediction in recent years, existing reviews have several limitations. First, most focus on technologies within a single disaster scenario, and do not provide a systematic overview of all five major disaster types. For instance, Xue et al. [7] and Mayank et al. [8] mainly focused on the use of machine learning for coal and gas outburst prediction, offering valuable technical insights but remaining limited to gas-related hazards. Similarly, Deng et al. [9] also focus solely on machine learning for predicting mine fires. This single-hazard focus makes it hard for readers to develop a comprehensive understanding of the technologies. Second, coal mine disasters are often linked by complex coupling mechanisms. Focusing on just one can overlook interconnections and hinder integrated prevention and control strategies. Single-hazard reviews also lack unified evaluation frameworks and benchmarks. Differences in indicators, datasets, and experimental settings make studies hard to compare or reproduce. Some indicators may also signal multiple disasters at once, making it difficult to systematically summarize these signals. This ultimately impedes the creation of integrated, cross-hazard early-warning systems.

To address the above issues, this paper provides a systematic review of recent advances in the application of artificial intelligence to the prediction of gas disaster, mine fires, mine water disaster, roof disaster, and coal dust disaster. From the three perspectives of technical mechanisms, application scenarios, and practical outcomes, we analyze the application pathways of AI methods across these five types of hazards. Furthermore, we summarize the indicator systems employed in different studies, compare the strengths and limitations of various algorithms in disaster prediction, and synthesize current research trends alongside the key unresolved problems. The overarching goal of this study is to establish a clear and comprehensive understanding of how AI technologies contribute to coal mine disaster risk prediction and prevention. Specifically, this paper aims to (1) identify the state of the art and key advances in AI-driven prediction models; (2) reveal commonalities and differences in data indicators, algorithmic mechanisms, and application outcomes across various disaster types; and (3) highlight the remaining challenges and potential pathways toward developing integrated, intelligent, and proactive disaster early-warning systems in coal mines. Finally, we propose future research directions worthy of in-depth exploration, with the aim of offering valuable references for subsequent studies.

**Figure 1:**
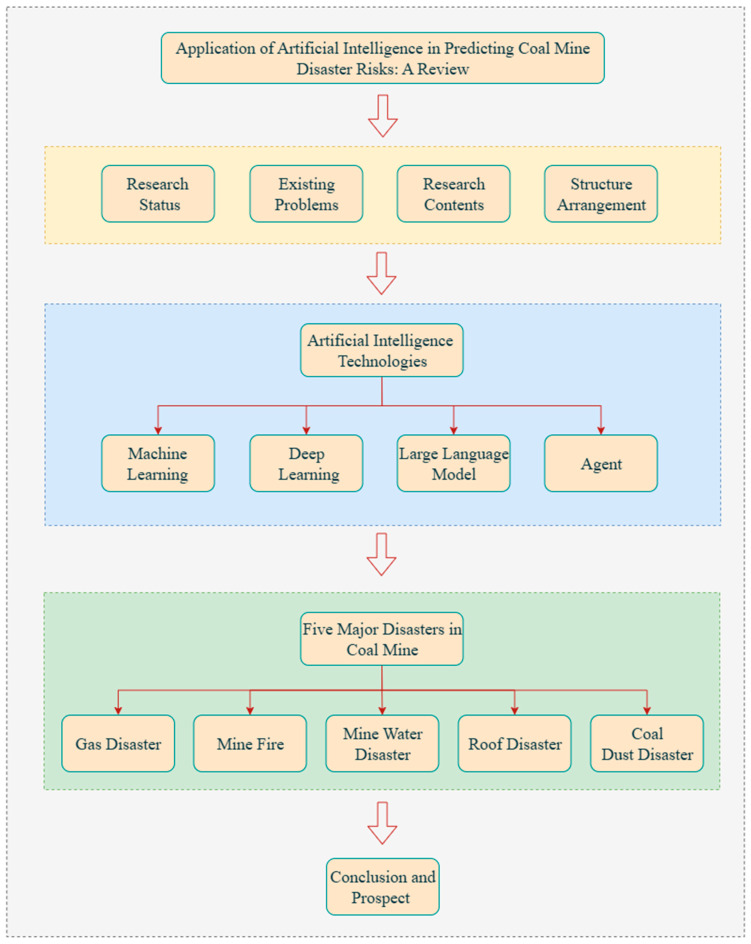
Framework of the review on the application of artificial intelligence in predicting coal mine disaster risks.

The structure of this paper is illustrated in Figure 1. Section 2 introduces relevant artificial intelligence techniques and outlines their underlying principles as well as current developments. Section 3 focuses on the five major types of disasters, namely gas disaster, mine fires, mine water disaster, roof disaster, and coal dust disaster, and reviews research progress, application cases, and comparative outcomes for each. Section 4 summarizes the cross-hazard challenges identified in existing studies and proposes future development directions together with key research topics.

## 2. Relevant Theoretical and Technical Foundations

Artificial intelligence (AI), as a frontier field of computer science, has achieved remarkable progress in areas such as image recognition, natural language processing, and machine learning [10]. Its core objective is to enable computers to simulate, augment, and even surpass human intelligence, thereby achieving perception, comprehension, and decision-making in relation to complex data. In the context of coal mine disaster prediction and prevention, the introduction of AI has not only expanded the methodological framework of research but also provided novel approaches for addressing disaster scenarios characterized by high risk and uncertainty.

In its early stages, AI primarily relied on rule-based expert systems, which suffered from limitations such as knowledge acquisition difficulties and poor adaptability. With advances in data collection and computational power, research gradually shifted toward machine learning (ML), which enables prediction and analysis by extracting patterns from historical data [11]. In recent years, deep learning (DL), supported by multi-layer neural network architectures, has effectively overcome the bottleneck of traditional machine learning that depended heavily on manually engineered features [12]. At the same time, emerging methods such as generative adversarial networks (GANs) [13] have demonstrated potential in augmenting samples and improving model performance under conditions of data scarcity. Entering the current stage, the advent of the Transformer architecture [14] and the development of large-scale models have accelerated the evolution of AI toward large language models (LLMs) and autonomous agents.

According to learning paradigms, machine learning can be classified into supervised learning, unsupervised learning, semi-supervised learning, and reinforcement learning [15,16]. In the field of coal mine disaster monitoring, supervised learning is the most widely used modeling approach, applied extensively to gas disaster, mine fires, mine water disaster, roof disaster, and coal dust disaster. Typical tasks include classification (e.g., risk level categorization), regression (e.g., hazard value estimation), clustering (e.g., disaster area partitioning), and anomaly detection [17]. Unsupervised learning is commonly employed for disaster area clustering and latent pattern discovery. Semi-supervised learning demonstrates advantages in scenarios with limited labeled samples, while reinforcement learning has been explored for optimizing mine production scheduling and emergency decision-making.

**Figure 2:**
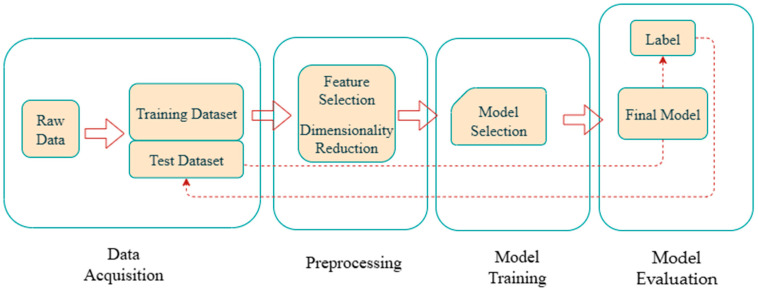
Workflow of machine learning model construction.

The workflow for constructing a machine learning model is illustrated in Figure 2 and typically consists of four key stages: data acquisition, preprocessing, model training, and model evaluation. Data serve as the foundation for model training. In coal mine disaster prediction, data sources mainly include online monitoring sensors, geological survey records, historical accident data, and video or image surveillance. Since raw data often contain missing values, anomalies, and noise, preprocessing techniques such as data cleaning and feature engineering are required to ensure reliability in subsequent modeling. Feature selection and feature extraction play a critical role in determining model performance. Feature selection methods are commonly employed to eliminate highly correlated variables, while techniques such as principal component analysis (PCA), gray theory, and multiple imputation are used for dimensionality reduction and feature representation optimization, thereby enabling the model to learn more efficiently. During the model training stage, different algorithms are selected depending on the task objectives. Table 1 summarizes commonly used algorithms, their application scenarios, and associated strengths and weaknesses. In the model evaluation stage, a series of metrics are typically applied to validate reliability. For classification tasks, accuracy, precision, recall, and the F1-score are frequently used to assess predictive performance. For regression tasks, mean squared error (MSE), mean absolute error (MAE), and the coefficient of determination ($R^{2}$) are commonly adopted to evaluate model fit. In addition, robustness and real-time performance must be considered to ensure that models can operate stably under the complex and dynamic conditions of underground coal mines.

**Figure 3:**
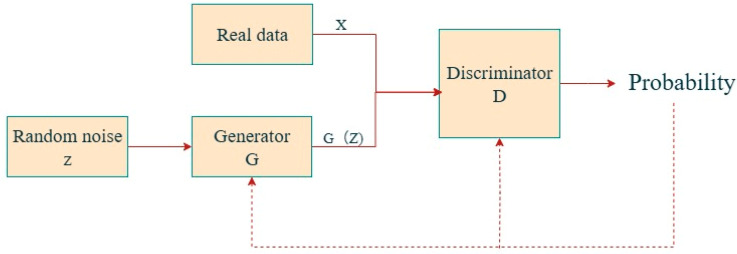
Structure of GAN. The generator *G* takes random noise *z* as input to generate “fake data” *G(z)*, while the discriminator *D* receives both real data x and fake data *G(z)* simultaneously and outputs the probability that the data is real. This “generation—discrimination” adversarial process is the core mechanism for GAN to achieve data generation.

At the model level, traditional machine learning approaches such as SVM and RF retain advantages in scenarios involving small samples and structured data, owing to their interpretability and relatively low computational cost. In contrast, deep learning models such as CNNs and LSTMs excel at handling high-dimensional, multimodal, and time-dependent monitoring data, which commonly arise in coal-mining operations. These models are particularly effective in addressing challenges related to multimodal data fusion, temporal feature modeling, and the characterization of disaster evolution patterns in monitoring [40,41,42]. GAN, as an emerging unsupervised learning framework, offers another promising solution. As illustrated in Figure 3, GAN can generate synthetic samples that approximate the distribution of real data, thereby alleviating issues of limited datasets and class imbalance in disaster monitoring [43,44].

Artificial intelligence provides multi-level technical support for coal mine disaster prediction through a systematic mechanism that spans data processing, feature representation, model construction, and intelligent decision-making. In data level, coal mine monitoring generates heterogeneous and high-frequency data from multiple sensors, including gas concentration, microseismic activity, temperature, humidity, and air velocity. AI-based preprocessing techniques—such as denoising autoencoders, attention-based filtering, and synthetic data generation (e.g., GAN or WGAN-GP)—enable noise reduction, missing-data imputation, and dataset balancing. These methods enhance the representativeness and stability of data, forming a solid foundation for subsequent modeling. In feature-learning level, traditional empirical features are often insufficient to capture nonlinear and multi-scale relationships among parameters. deep-learning models, such as CNN, LSTM, and autoencoder, can automatically learn discriminative latent features from raw signals, identifying hidden precursors of disasters (e.g., gas emission anomalies or sudden stress fluctuations). Feature fusion and attention mechanisms further improve feature interpretability and relevance across different data modalities. In spatio-temporal modeling level, the evolution of coal mine disasters exhibits significant temporal dependencies and spatial coupling characteristics. Advanced models such as temporal convolutional networks (TCNs), graph neural networks (GNNs), and Transformer architectures effectively capture multi-dimensional correlations among monitoring nodes. These models support dynamic risk assessment by describing both the propagation of hazardous factors and their temporal evolution trends. These AI-driven mechanisms not only improve the accuracy and timeliness of coal mine disaster prediction but also promote the transformation of traditional experience-based management into a data-driven, adaptive, and intelligent prevention paradigm.

**Figure 4:**
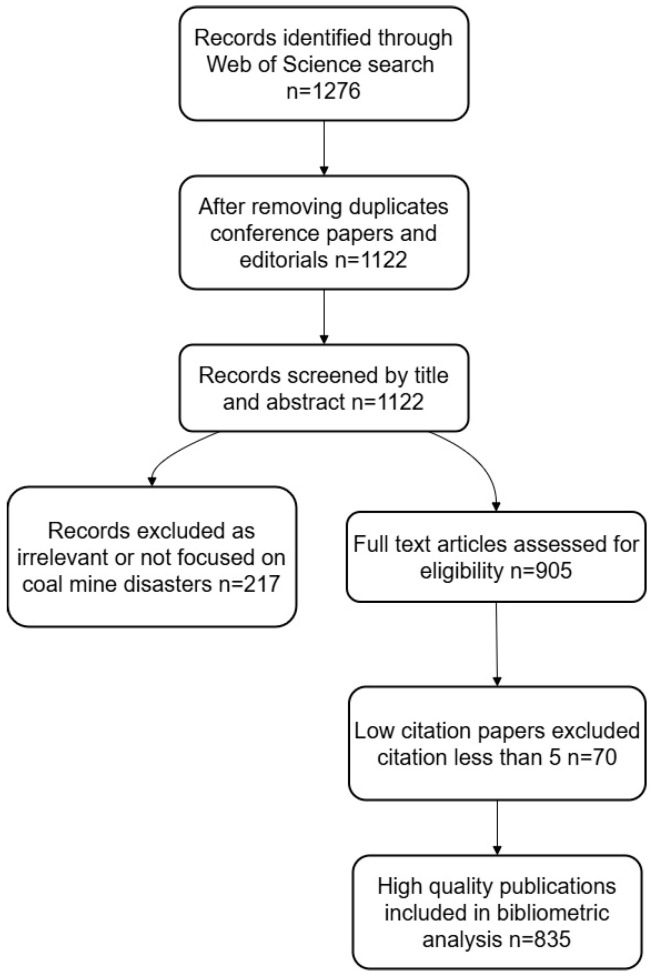
Literature Screening Flowchart.

**Figure 5:**
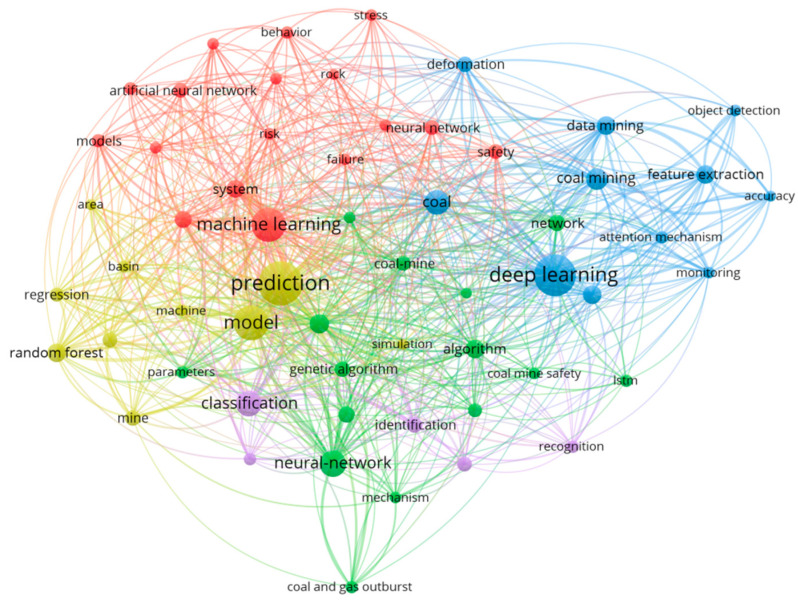
Keyword co-occurrence map of artificial intelligence in coal mine disaster.

To further explore the current research priorities and technological trends of AI in coal mine disaster prediction, this study performed a comprehensive bibliometric retrieval and analysis based on the Web of Science (WoS) Core Collection database. The specific flowchart is shown in Figure 4. The search was conducted on 15 July 2024, with the following query string applied to the “Topic” field (including title, abstract, author keywords, and Keywords Plus): TS = (“coal mine” AND disaster AND prediction AND (“artificial intelligence” OR “machine learning” OR “deep learning” OR “neural network” OR “intelligent system”)). The search was limited to the period from January 2013 to June 2024, and only journal articles and review papers written in English were included. Conference papers, editorials, and duplicates were excluded. A total of 1276 records were initially retrieved. After removing irrelevant and low-citation papers (less than five citations) and those unrelated to coal mine disaster prediction, 835 publications were retained as “high-quality” publications. The bibliometric analysis was conducted using VOSviewer (version 1.6.20). The overall screening and selection process is summarized in Figure 5.

In the visualization, the size of each node indicates the frequency of keyword occurrence, the thickness of the connecting lines reflects the strength of co-occurrence, and the colors represent different clusters. As shown in the keyword co-occurrence map, current research mainly focuses on the following areas: model optimization and risk modeling (red), image recognition and data-driven prediction (blue), model generalization and performance evaluation (yellow), algorithm integration and intelligent control (green), and recognition and classification tasks (purple). Among these, keywords such as machine learning, deep learning, and prediction appear with the highest frequency, underscoring that artificial intelligence has become the core tool in coal mine disaster research. This also demonstrates a clear shift in focus from traditional statistical approaches toward data-driven intelligent algorithms.

In recent years, the rapid development of LLMs has propelled AI into a new stage. Centered on the Transformer architecture, LLMs acquire powerful capabilities in language understanding and generation after pretraining on massive datasets [45]. The emergence of ChatGPT and similar models has triggered a worldwide surge in foundation model development [46]. Depending on application domains, LLMs can be categorized into general-purpose foundation models and industry-specific vertical models [47]. Foundation models, pretrained on large-scale general-purpose corpora, demonstrate strong generalization and transferability across tasks, enabling efficient adaptation to new problems even with limited labeled data and substantially reducing the reliance on manual feature engineering [48]. Notable representatives include OpenAI’s GPT series [49], Meta’s LLaMA [50], China’s DeepSeek [51], and Alibaba’s QWen model. Despite their success, the deployment of foundation models faces challenges in terms of substantial computational demands, high training costs, and limited domain-specific knowledge. To address these limitations, researchers have increasingly turned toward domain-specific vertical LLM, which are pretrained and optimized using high-quality, expert-curated data in targeted application areas [52]. In the mining sector, such exploration remains at an early stage. For example, the Pangu Mining Model, jointly developed by Shandong Energy Group and Huawei, represents the first commercially applied large-scale AI model in mining worldwide [53]. Similarly, the YUKON Mining Model, led by the China University of Mining and Technology (Beijing), integrates the full lifecycle of mining operations to achieve comprehensive monitoring and intelligent decision-making. The Sunstone Mining Model, developed by the China Coal Technology & Engineering Group, highlights safety and emergency management by integrating multimodal datasets, including 50 billion safety monitoring records, three million visual images, 20 specialized journals, and 20 billion words of technical documents [54]. These models, designed for mine safety and emergency management, combine multimodal data with domain knowledge and demonstrate promising application prospects [55].

Looking ahead, the development of domain-specific vertical LLM is expected to evolve toward four major directions. First, multimodal fusion and embodied intelligence will become the core, enabling the integration of vision, language, sound, and sensor data to form a unified cognitive system capable of complex spatiotemporal reasoning and perception-based decision-making. Second, lightweight and efficient training paradigms—such as parameter-efficient fine-tuning, knowledge distillation, and retrieval-augmented generation—will reduce computational and data requirements, allowing vertical models to be rapidly deployed on edge devices in mines. Third, self-evolving and continuously learning mechanisms will enable models to update themselves with newly collected field data, ensuring adaptability to dynamic mining environments and evolving safety patterns. Finally, trustworthy and interpretable AI frameworks will become essential to address issues of model opacity, bias, and safety validation, especially in high-stakes domains such as coal mine disaster prediction and emergency response. Together, these trends will transform vertical models from static expert systems into autonomous, adaptive, and transparent intelligent agents capable of supporting real-time risk prediction and decision-making in mining operations.

At the same time, the introduction of agents has provided new opportunities for the practical implementation of LLMs. Agents are characterized by autonomous perception, reasoning, learning, and execution, enabling them to accomplish complex tasks in dynamic environments. Building upon this, multi-agent systems (MAS) coordinate multiple agents to achieve closed-loop management that encompasses data collection, feature extraction, predictive inference, and emergency response [56].Although applications of MAS in the mining domain are still at an early stage, several pioneering studies have already emerged. For instance, Sun et al. applied MAS to intelligent decision-making for gas states, which significantly improved the efficiency of gas alarm handling in coal mines [57]. Similarly, Cao et al. developed a MAS-based model (XCoalChat) for coal mine equipment maintenance by leveraging the extensive body of existing maintenance knowledge [58]. These studies suggest that MAS holds great potential in the field of mine safety. Through collaborative interactions among agents, MAS can integrate heterogeneous sources of information, optimize decision-making processes, and provide new technological pathways for accurate disaster prediction and timely response in coal mines [59].

In summary, the development of AI technologies has progressed from traditional ML to DL, and more recently to LLMs and agents. ML, with its interpretability and stability, laid the foundation for data-driven modeling. DL overcame the limitations of hand-crafted features through end-to-end feature learning, demonstrating clear advantages in handling high-dimensional, complex, and multimodal data. LLMs, built upon massive pretraining and cross-task transfer capabilities, are propelling AI from task-driven to knowledge-driven paradigms. Meanwhile, agents, particularly MAS, have introduced new approaches to achieving closed-loop processes that integrate perception, reasoning, and decision-making. Collectively, these advances suggest that AI is gradually evolving into a comprehensive ecosystem composed of multi-layered and multi-faceted technologies. Building on this foundation, the following sections systematically analyze the application and progress of AI methods in addressing five typical categories of coal mine disasters, with a focus on identifying their strengths and limitations in prediction, early warning, and emergency response.

## 3. Application of Artificial Intelligence in Predicting Major Disaster Risks in Mines

This section specifically focuses on coal mines, rather than on the mining industry in general. This definition aligns with the overall objective of the present study—to synthesize and evaluate the applications of artificial intelligence (AI) in predicting and managing the major disaster risks inherent to coal mining operations, particularly within the context of China’s deep and complex mining environments.

To establish a coherent foundation for the subsequent analysis, a brief integrative overview of the five major categories of coal mine disasters is provided. These disasters represent the most frequent and destructive types of hazards threatening mine safety, and they are inherently interrelated through shared geological, mechanical, and environmental mechanisms. Specifically: (1) coal and gas outburst, characterized by the sudden and violent ejection of gas and coal, posing severe threats to miners’ safety; (2) mine fire, resulting from spontaneous combustion or ignition of flammable materials within underground environments; (3) mine water disaster, caused by the uncontrolled inflow or accumulation of groundwater or surface water into mine workings; (4) roof disaster, involving the collapse or instability of rock strata in the mine roof that endangers structural integrity; and (5) coal dust disaster, associated with the suspension and explosion of fine coal particles. These five categories collectively illustrate the multi-factor and multi-scale nature of coal mine disaster formation and propagation. Their complex physical and temporal coupling significantly increases the difficulty of early detection and prediction. Consequently, applying AI-based models offers a promising pathway to integrate heterogeneous monitoring data, capture nonlinear hazard evolution patterns, and achieve more intelligent and proactive disaster prevention.

The following subsections systematically review AI applications across these five disaster types, analyzing representative models, data sources, and predictive strategies while identifying current limitations and potential research frontiers.

### 3.1. Gas Disaster

**Figure 6:**
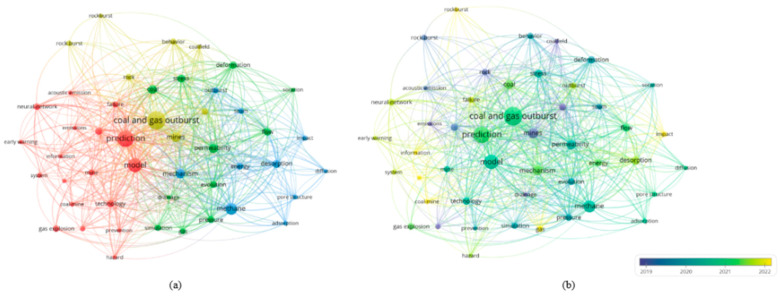
Research focus and temporal evolution of gas disaster prediction with AI. (**a**) Keyword co-occurrence clustering map based on 218 publications from the Web of Science Core Collection. (**b**) Temporal distribution of research hotspots, the average publication year of the papers containing those keywords.

Gas is one of the most critical factors that cause disasters in coal mining. Among all types of coal mine accidents, those caused by gas account for the highest proportion, the highest mortality, and the most serious economic losses. These accidents mainly include coal and gas outbursts, gas combustion, gas suffocation, and gas explosions. In China, more than one-third of coal mines are classified as high-gas or outburst-prone, which brings enormous pressure to safe production [60]. To analyze research trends in gas disaster prediction, relevant publications were retrieved from the Web of Science Core Collection using the following search query: TS = (“coal and gas outburst” OR “gas disaster” OR “gas emission” OR “gas prediction”) AND (“artificial intelligence” OR “machine learning” OR “deep learning”). The search covered the period from January 2013 to June 2024 and included only peer-reviewed journal articles and reviews written in English. Conference papers, notes, and articles not directly related to underground coal mining were excluded. After screening for topic relevance and minimum citation thresholds (≥5 citations), 218 publications were included in the analysis. The bibliometric visualization was generated using VOSviewer to identify key research clusters and development trends in gas disaster prediction. A keyword co-occurrence cluster analysis was then conducted using VOSviewer. As shown in Figure 6a, current research mainly focuses on four areas: the development of predictive models and intelligent early-warning systems (red cluster), the modeling of outburst mechanisms (green cluster), the study of gas migration patterns (blue cluster), and the simulation of catastrophic processes (yellow cluster). The keywords prediction, model, and technology form frequent connections across clusters, reflecting a shift in research interest from mechanistic studies toward intelligent modeling and system integration. The temporal distribution presented in Figure 6b shows that before 2019 (purple), studies were mainly devoted to outburst mechanisms. Since 2020, machine learning has been introduced to construct gas concentration prediction models for engineering applications. After 2022 (yellow), research hotspots shifted toward intelligent early-warning platforms and multisource data integration, showing an evolution from tool-based methods to systematic and platform-oriented approaches. Coal and gas outbursts, as one of the most dangerous and complex dynamic disasters, are characterized by sudden occurrence and destructive power. They often lead to severe casualties and heavy property losses [61]. Moreover, they are closely associated with other accidents caused by gas, such as explosions and suffocation. Therefore, effective monitoring and prediction are not only essential for ensuring production safety but also critical for preventing a chain of gas-related accidents.

Traditional approaches for predicting coal and gas outbursts mainly include empirical methods, indicator-based methods, and geophysical exploration. These approaches rely heavily on geological exploration data and expert knowledge. However, due to the complex and variable underground environment, coal and gas outbursts are influenced by multiple interrelated factors with highly nonlinear relationships, making it difficult for traditional methods to accurately capture real situations [62]. In recent years, with the rapid development of AI, an increasing number of studies have shifted toward risk modeling and prediction using ML and DL techniques. Compared with conventional statistical models, AI demonstrates clear advantages in handling nonlinear, multisource, and unstructured data. In practice, algorithms such as SVM, RF and ensemble learning have been widely applied to evaluate and predict the risk level of outbursts [63].

The primary challenge in predicting coal and gas outbursts lies in the complexity and uncertainty of the data. The triggering factors involve heterogeneous information derived from coal properties, gas migration, and ground stress conditions [7]. To build a ML model with reliable classification performance and strong generalization ability, the fundamental requirement is to ensure that the input data are of high quality and representative. Table 2 summarizes numerous indicators related to coal and gas outbursts. Some of these indicators exhibit significant correlations, and directly inputting them into the model may lead to an exponential increase in computational cost and adversely affect model stability [64,65]. To address this issue, researchers have primarily adopted feature engineering strategies, applying methods such as PCA, gray theory, and rough set (RS) analysis for dimensionality reduction and feature selection. Wang et al. [66] applied PCA to extract three dominant influencing factors from ten commonly used coal seam indicators, thereby retaining the major information while improving the convergence speed and stability of model training. Hu et al. [67] employed gray relational analysis to rank the importance of variables such as gas content and coal firmness, providing a quantitative basis for feature selection and effectively reducing the influence of redundant attributes. To tackle the challenges of scarce and incomplete accident samples, Zheng et al. [68] were the first to introduce the multiple imputation algorithm to process outburst accident data. By reasonably supplementing missing variables, they expanded the effective sample size and significantly enhanced both the robustness and practicality of the model. Collectively, these studies demonstrate that rational dimensionality reduction, correlation analysis, and missing data imputation provide high-quality inputs for ML and DL models, thereby improving prediction performance and application value.

It is noteworthy that the indicators summarized in Table 2 vary widely in their quantitative ranges due to differences in geological conditions, coal types, and measurement methods across studies. For instance, the burial depth of seams prone to outburst is typically greater than 600–800 m, while the gas content often exceeds 8–12 m^3^/t, which is considered a critical threshold for outburst-prone seams in China. Similarly, the gas pressure usually ranges from 0.7 to 1.5 MPa in hazardous areas, and the coal firmness coefficient tends to be below 0.5–0.8, indicating a soft coal structure that facilitates gas release. However, these threshold values are not universal; variations can be attributed to regional geological differences, the adsorption capacity of coal, and experimental methods such as low-field NMR versus mercury intrusion for porosity measurement. Therefore, while these indicators provide a consistent framework for assessing outburst risks, further standardization and cross-comparison among datasets are essential to establish reliable benchmarks and improve model generalizability.

The selection and construction of predictive models constitute the core of coal and gas outburst forecasting systems. In recent years, a variety of ML and DL models have been introduced to enhance both accuracy and robustness [8]. Traditional ML methods still play an important role, especially in scenarios with limited samples and high requirements for interpretability. SVM was widely applied in the early stages due to their stability in handling high-dimensional and nonlinear data. However, with the increasing volume of monitoring data, their training cost and storage overhead have significantly increased. To address these limitations, You et al. [18] innovatively combined t-distributed Stochastic Neighbor Embedding (t-SNE) with Genetic Algorithms (GA) to optimize SVM, classifying the outburst risk into four severity levels. Similarly, Wang et al. [69] proposed a transformer combined with SVM model, where Permutation Feature Importance (PFI) was used to assess the contribution of input variables. Their results confirmed that gas content, desorption quantity, and coal desorption rate were the most critical factors. Tree-based models have gradually gained adoption in mine disaster prediction due to their intuitive decision paths and robustness in handling missing data [70]. Decision trees, as the basic form, provide high interpretability, but their predictive accuracy is limited and often inadequate for high-risk scenarios [71]. To overcome this, ensemble tree methods such as Random Forest [72], Gradient Boosted Decision Trees [73], and their advanced variants, including XGBoost [26] and LightGBM, have been widely employed. These models improve adaptability to complex mining datasets through algorithmic enhancements and engineering optimizations. For example, Zheng et al. [26] integrated XGBoost with MI to expand the sample size, effectively mitigating the problem of data scarcity and enhancing both recognition capability and robustness of the model.

**Figure 7:**
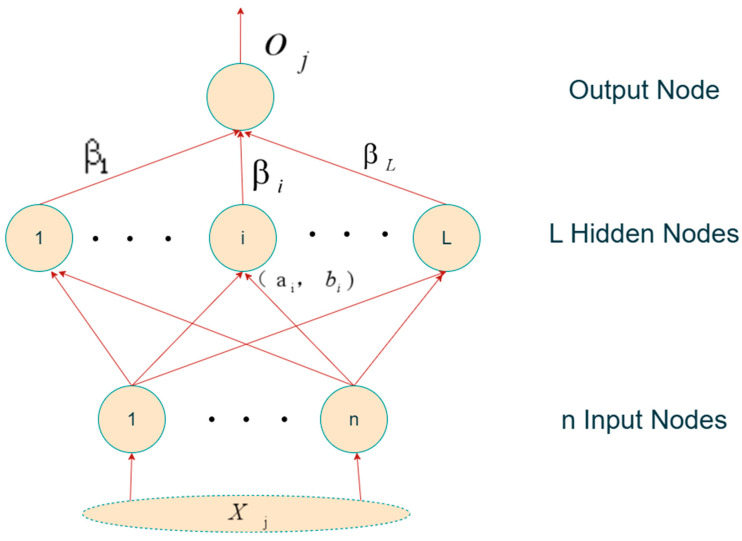
Extreme Learning Machine network structure diagram. This diagram illustrates the architecture of a neural network model. There are *n* input nodes that receive input data $X_{j}$. These input nodes are connected to *L* hidden nodes, with each hidden node *i* characterized by parameters $\left(a_{i},b_{i}\right)$. The hidden nodes then connect to an output node $O_{j}$, and the connections between hidden nodes and the output node are weighted by $\beta_{1}$, $\beta_{i}$, $\beta_{L}$.

Traditional ML methods exhibit strong performance in specific scenarios, but their reliance on manually designed features and limited capacity to capture complex nonlinear relationships have gradually shifted research focus toward neural networks with autonomous feature learning ability. BPNN possess powerful nonlinear fitting and self-learning capabilities, yet they also suffer from slow training speed and susceptibility to local optima. To overcome these shortcomings, researchers have introduced optimization algorithms such as Particle Swarm Optimization (PSO), Genetic Algorithms (GA), and Simulated Annealing (SA). For instance, Wu et al. [74] combined GA and SA to optimize the weight parameters of BPNN, while Zhu et al. [27] applied Rough Set (RS) theory for dimensionality reduction and further incorporated GA optimization, thereby achieving collaborative optimization of feature selection and model training. Extreme Learning Machine (ELM) and its improved variant, Kernel ELM (kELM) [75], have emerged as promising tools in prediction research due to their fast training speed and strong generalization ability [76], Figure 7 illustrates the network architecture of ELM. Building upon this, Miao et al. integrated the Whale Optimization Algorithm (WOA) to dynamically regulate the weights and biases of ELM, and further combined Case-Based Reasoning (CBR) to provide risk-mitigation recommendations based on predictive results [77,78]. Although such optimization techniques alleviate the local optimum problem of shallow neural networks to some extent, their ability to represent highly complex outburst phenomena remains limited, which motivates the adoption of deep learning approaches.

**Figure 8:**
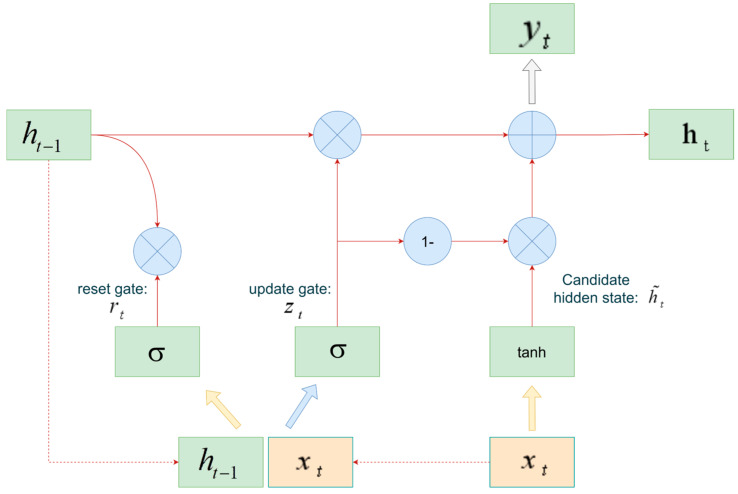
GRU structure diagram. It consists of a reset gate $r_{t}$ and an update gate $z_{t}$, both computed via the sigmoid function σ using the current input $x_{t}$ and previous hidden state $h_{t-1}$. The reset gate determines how much past information to forget, while the update gate controls the flow of information into the current hidden state $h_{t}$.

DL models, with their capability for automatic feature extraction and advanced spatiotemporal modeling, have gradually become the mainstream techniques in this field [79]. Acoustic Emission (AE) and Electromagnetic Radiation (EMR), often regarded as precursory information of coal-rock fracture, are widely applied in outburst prediction [80,81]. CNN has been employed for the image-based recognition of AE and EMR signals, enabling hierarchical feature learning directly from raw data. However, under the high-noise conditions of underground coal mines, signal attenuation remains severe, which restricts practical deployment [82]. To address this limitation, Liu et al. [83] introduced a semi-supervised classification approach that jointly utilizes a small set of labeled samples and a large volume of unlabeled data. This method reduces reliance on manual annotations and enhances the detection of latent risks. As a simplified variant of LSTM, the GRU significantly reduces parameter size and computational cost while retaining the ability to capture long-term dependencies, making it more suitable for real-time monitoring scenarios in coal mines. Its architecture is illustrated in Figure 8. Leveraging these advantages, Wen et al. [38] optimized GRU parameters using a combination of PSO and GA, and further integrated the model with Spark Streaming to realize real-time prediction and early warning of gas concentration. The system achieves a response time of less than 8 s, effectively shifting risk management from post-event remediation to proactive intervention. In summary, DL has substantially improved prediction accuracy and adaptability. Nevertheless, its heavy reliance on large-scale, high-quality datasets and limited robustness in complex environments remain critical challenges.

In recent years, the emergence of LLMs has opened new avenues for systematic and intelligent approaches to predicting coal and gas outbursts. Du et al. [84] integrated LLMs with Bayesian networks to achieve causal inference and risk-oriented decision-making under incomplete information, thereby improving the scientific rigor and interpretability of prediction models. Wang Guofa et al. [85] applied transparent geological modeling and digital twin technology to incorporate three-dimensional geological structures, mine ventilation, and gas migration patterns into a visualized simulation framework, enabling real-time disaster evolution and early warning. Similarly, Wang [86] introduced digital twin technology into outburst prediction by coupling physical and virtual entities through an airflow control system. By combining sub-dimension evolutionary particle swarm optimization (sdPSO) with a quantum gate node neural network (QGNN), the framework realized dynamic data mapping and interaction between physical and virtual spaces, which alleviates the challenges of data scarcity and hazardous sample collection. Furthermore, Sun et al. [57] proposed a distributed multi-agent system that integrates monitoring, analysis, and decision-making into a unified platform. Within this framework, individual agents coordinate through shared knowledge bases and rule libraries to achieve task allocation and collaboration, demonstrating the potential for end-to-end automation. Overall, these approaches show promising outcomes in proof-of-concept studies and localized applications. Nevertheless, most remain at the experimental or pilot stage, and further validation is required to ensure their scalability and reliability under complex underground mining conditions.

As summarized in Table 3, research progress in coal and gas outburst prediction demonstrates a clear evolution from traditional feature-engineering-based models toward multi-modal intelligent frameworks centered on DL and LLM. This paradigm shift has markedly improved prediction accuracy, timeliness, and system-level integration [87]. However, existing research still faces challenges such as inconsistent and non-transparent indicator systems, insufficient model generalization and transferability across mines, and disconnect between prediction outcomes and emergency dispatch systems. Future efforts should focus on establishing standardized multidimensional indicator systems, implementing cross-mine multimodal modeling, and developing intelligent decision-making agent systems to build a scientific, interpretable, and implementable closed-loop prediction and prevention system covering the entire process.

### 3.2. Mine Fire

**Figure 9:**
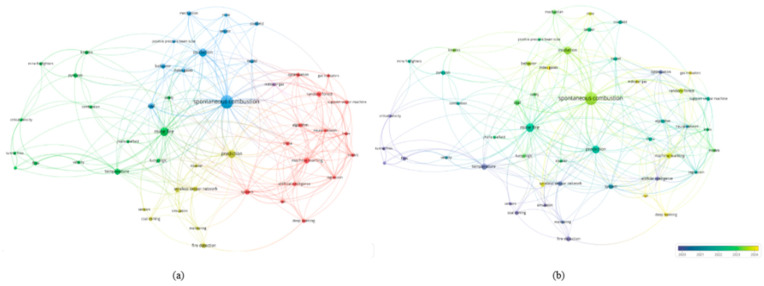
Research focus and temporal evolution of mine fire studies with AI. (**a**) Keyword co-occurrence clustering map based on publications from the Web of Science database. (**b**) Temporal distribution of research hotspots.

Mine fires represent one of the most severe hazards in coal mining, directly threatening the safety of workers and frequently triggering secondary disasters such as gas and coal dust explosions, which further amplify the destructive impact. Compared with gas accidents, mine fires are characterized by sudden onset, rapid spread, and strong concealment. Traditional approaches relying on sensor alarms and expert judgment often exhibit significant delays in early detection and rapid response. Accident statistics reveal that between 2000 and 2023, a total of 595 major coal mine accidents occurred in China, of which 360 were fire accidents, accounting for more than 60% and resulting in 8483 fatalities [89]. These figures highlight the high frequency and fatality of fire hazards within the spectrum of coal mine disasters, underscoring the urgent need for more effective prediction and early-warning methods. Against this background, AI has gradually emerged as an important breakthrough in mine fire research. To systematically capture research frontiers and evolutionary trends, a keyword co-occurrence network was constructed based on the Web of Science database, and the co-occurrence map (Figure 9a) and the average publication year overlay map (Figure 9b) were generated using VOSviewer. As shown in Figure 9a, research hotspots are mainly centered on spontaneous combustion, and mine fire, reflecting the long-standing focus of the academic community on fire mechanisms, monitoring, and propagation processes. At the same time, keywords such as prediction and system establish links between fire dynamics and intelligent modeling, suggesting that artificial intelligence methods have been increasingly incorporated into fire risk prediction. The temporal evolution in Figure 9b further indicates a shift in focus, moving from early studies of physical processes and fire dynamics toward mechanism-oriented and monitoring-related topics. Since 2023, artificial intelligence technologies have appeared with growing frequency and prominence, signaling that data-driven intelligent modeling is gradually replacing purely mechanism-based approaches and becoming the central paradigm of current research.

Based on causative factors, mine fires can be categorized into externally triggered and internally originated fires [90]. Externally triggered fires are primarily caused by electrical short circuits, welding sparks, excessive friction from machinery, or unauthorized open flames [91], and are characterized by sudden onset with clearly identifiable external triggers. Traditional monitoring approaches, which often rely on single sensors or image recognition, frequently encounter delayed responses or significant detection errors under underground conditions with low lighting, dust, and noise. To overcome these limitations, Zhao et al. [92] improved the YOLOv5s model by incorporating a dynamic weighting mechanism that fuses video detection data with sensor signals such as temperature, smoke, and CO levels, significantly enhancing both real-time performance and robustness in complex environments. Chen et al. [93] addressed the challenge of early fire prediction on conveyor belts by developing a CNN-LSTM hybrid model, where the CNN captures spatial features and the LSTM models temporal dependencies of fire-related parameters, enabling the prediction of key combustion metrics such as heat release rate and mass loss rate. These studies indicate that monitoring of externally triggered fires is gradually shifting from single-sensor approaches toward multimodal data integration and intelligence-driven algorithms.

In contrast, internally originated fires primarily result from spontaneous combustion of coal or oxidation reactions of combustible materials under specific underground conditions [94]. These fires are highly concealed and develop slowly, making traditional identification methods that rely on expert judgment often delayed and susceptible to subjective bias. The spontaneous combustion process is influenced by multiple interacting factors, including coal properties, oxygen availability, temperature, and humidity, which significantly increases the complexity of prediction compared with externally triggered fires [95]. In recent years, researchers have increasingly applied ML and DL techniques to leverage historical monitoring data for uncovering latent nonlinear features and temporal patterns. Table 4 summarizes commonly used indicators for predicting coal spontaneous combustion, which are often highly non-stationary and prone to sudden changes, placing stringent requirements on model robustness and temporal modeling capability. To more effectively identify potential risks and enhance the intelligence of early warning systems, an increasing number of studies have employed artificial intelligence models for pattern recognition and trend prediction of mine fire indicators, thereby enabling more efficient disaster forecasting.

As shown in Table 4, gas concentration indicators for predicting coal spontaneous combustion exhibit characteristic quantitative ranges that vary depending on the oxidation stage and environmental conditions. In the early stage of oxidation, the CO concentration typically remains below 10 ppm, increasing rapidly once temperature exceeds 70–90 °C, serving as an early warning sign. The $O_{2}$ concentration correspondingly decreases from the normal atmospheric level (≈20.9%) to below 18% in enclosed areas, reflecting active oxidation. Meanwhile, the $CO_{2}$ concentration generally increases above 1%, indicating intensified combustion reactions. The presence of $C_{2}H_{4}$(ethylene) becomes evident only at temperatures above 110 °C, marking the transition from low-temperature oxidation to pyrolysis, while $C_{2}H_{6}$ (ethane) often appears at even higher temperatures (>150 °C), representing secondary oxidation of methane. Variations in these thresholds across studies arise from differences in coal rank, ventilation rate, and the analytical precision of instruments such as gas chromatography and mass spectrometry. Therefore, combining both qualitative gas composition analysis and quantitative concentration benchmarks enables a more accurate identification of spontaneous combustion stages and improves early-warning reliability.

In the early warning of mine fires, the variation trend of coal temperature is the most direct and sensitive precursor. Continuous monitoring of coal seam temperature allows the detection of weak heat accumulation in the initial stage of spontaneous combustion, and, when combined with the rate of temperature rise and its spatial distribution, provides a reliable basis for determining the fire development stage and potential spread. This information is essential for early warning grading, ventilation adjustment, and proactive intervention [19]. However, large-scale real-time temperature monitoring in underground coal seams is difficult to achieve, and researchers generally rely on modeling approaches for indirect prediction. Studies have demonstrated that oxygen consumption and CO concentration are the most sensitive and stable oxidation markers, showing a sustained increase with rising coal temperature. Meanwhile, gases such as $CO_{2}$, $CH_{4}$ and $C_{2}H_{4}$ are released more rapidly during medium- and high-temperature stages, exhibiting distinct phase-dependent variation patterns [96]. The dynamic evolution of these gas concentrations effectively reflects the oxidation stage of coal, forming the core foundation for temperature prediction and fire trend assessment [9]. Based on this mechanism, ML has been increasingly introduced to address the highly nonlinear mapping between gas concentration and coal temperature [97,98]. For instance, Wang et al. [99] proposed an SSA-CNN model that integrates singular spectrum analysis with CNN, enabling effective extraction of critical gas concentration features and capturing their nonlinear relationship with coal temperature, particularly achieving higher accuracy during the rapid heating stage. In parallel, some studies have focused on predicting coal spontaneous combustion from physicochemical parameters. Zhao et al. [100] employed BPNN to reveal the influence of activation energy, porosity, and moisture content on coal spontaneous combustion tendency, providing new insights into the mechanism from the perspective of intrinsic coal properties and supporting the design of targeted prevention measures. Nevertheless, models driven by single-feature inputs remain insufficient to capture the complexity of spontaneous combustion mechanisms, which has prompted a gradual shift toward multi-source data fusion.

Beyond coal temperature prediction, recent research has gradually extended toward higher-level intelligent applications, including multi-source information fusion, intelligent classification, risk level identification, and temporal modeling. Wang et al. [101] constructed a multi-source feature framework that integrates gas concentration, ventilation parameters, and temperature information, and compared multiple ML algorithms to identify the optimal pathway for fire recognition. At the perception layer, Kong et al. [102] developed an enhanced YOLOv8s model for real-time detection of fire and smoke. By introducing multi-scale feature extraction and an attention mechanism, the model strengthened the representation of color, texture, and spatial distribution features in images, thereby maintaining high detection accuracy even in underground environments characterized by poor illumination and dust interference. At the decision-making layer, Kamran et al. [25] combined GBDT and LightGBM to establish a fire intensity classification model. Based on the Graham ratio calculated by Equation (1), fire intensity was divided into six levels: Normal fire (≤0.4), Emergency fire (0.5), Asertive fire (>0.5<1), Progressive fire (>1<2), Active fire (>3), Intensive fire (≥7). This quantitative framework provides a systematic basis for early risk-level warnings and the formulation of preventive measures. Overall, these approaches represent a progressive evolution from multi-source feature integration to real-time perception and intelligent classification-based decision-making. However, the predictive performance of such models remains highly dependent on the completeness and quality of input data, which underscores the potential value of integrating large-scale AI models and intelligent agent-based systems in future studies.

(1) $$\mathrm{Graham}’\mathrm{sratio}=100\times \frac{\mathrm{CO}}{\left(0.265\times N_{2}-O_{2}\right)}$$

**Figure 10:**
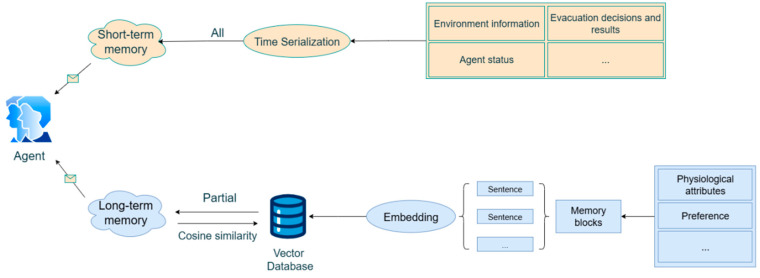
Design of agent’s long-term and short-term memory. Information including environment details, evacuation decisions and results, and Agent status undergoes time serialization and is fully stored in the Agent’s short—term memory. For long—term memory, sentences are embedded to form a vector database. Partial content from this database, selected via cosine similarity, is stored in the Agent’s long—term memory. The Agent also incorporates elements like physiological attributes and preferences, which interact with memory blocks.

In recent years, the emergence of LLM and AI agent technologies has opened new avenues for mine fire prediction and prevention. To address the high dimensionality, multi-parameter coupling, strong interactions, and pronounced temporal variability inherent in the coal spontaneous combustion process, Deng et al. [103] developed an intelligent early warning system based on LLMs. The system adopts a three-layer architecture consisting of intelligent perception, data transmission, and analytical decision-making, thereby enabling early detection, rapid response, and precise intervention, and providing a practical prototype for the intelligent management of mine fires. Meanwhile, Dang et al. [104] proposed a multi-agent fire evacuation simulation framework powered by LLMs. As illustrated in Figure 10, agents were designed with both short-term and long-term memory, allowing them to generate dynamic evacuation strategies under complex underground conditions. This approach enhances the scientific validity and operability of emergency response, offering an innovative solution for behavior modeling and rescue optimization in challenging mine environments. Collectively, these studies indicate that fire prediction is undergoing a transition from traditional parametric models and multi-source data fusion toward large model-driven, system-level intelligent governance. This reflects a clear technological trajectory evolving from single-point monitoring to multimodal perception and, ultimately, to coordinated intelligent decision-making.

Table 5 summarizes the research progress of AI in mine fire prediction. It is evident that AI technologies have facilitated the evolution of disaster monitoring from single-indicator assessments to multi-source sensing and intelligent risk identification, gradually shaping a preliminary closed-loop framework that integrates multi-source perception, knowledge reasoning, and multi-agent decision-making. Nevertheless, several limitations remain. Current studies often suffer from the absence of standardized data protocols, insufficient model transferability across diverse geological conditions, reliance on limited predictive indicators, and inadequate alignment between algorithms and monitoring equipment. Looking ahead, the development of systematic, reliable, and deployable intelligent fire prediction systems may be achieved through the establishment of shared multi-mine databases, the adoption of federated learning, and the integration of multimodal data fusion, enabling more robust performance and cross-scenario adaptability.

### 3.3. Mine Water Disaster

**Figure 11:**
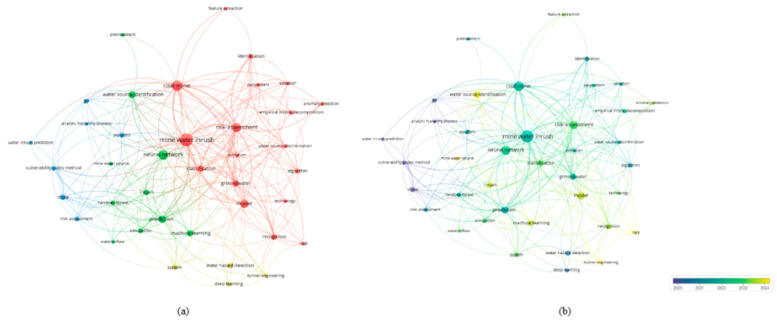
Research focus and temporal evolution of mine water disaster with AI. (**a**) Keyword co-occurrence clustering map based on publications from the Web of Science database. (**b**) Temporal distribution of research hotspots.

Mine water disaster is among the most severe and complex natural hazards in coal mining, posing a substantial threat to mine safety and sustainable production [107]. Mine water disasters primarily manifest in the forms of inrush, flooding, and inundation, and are characterized by sudden onset, high destructiveness, and the difficulty of rescue operations [108]. Once triggered, they not only damage equipment and infrastructure but also directly endanger the lives of mine workers. In regions with strong aquifer recharge and complex hydrogeological structures, water hazards can further disturb the regional hydrological system, leading to groundwater contamination and ecological degradation. According to the statistics of the National Mine Safety Administration, from 2000 to 2022, a total of 1206 coal mine water disasters occurred in China, resulting in 5018 fatalities. Among these, 103 were classified as major accidents, causing 2039 deaths [109]. Such figures highlight the severe economic and social consequences associated with mine water hazards. Against the backdrop of limited effectiveness of conventional prevention and control measures, the development of efficient monitoring and accurate prediction approaches has become a central challenge for safe and intelligent mining. Based on the keyword co-occurrence map (Figure 11a) and the overlay visualization by publication year (Figure 11b) generated with VOSviewer, current research on the application of AI in mine water hazard prediction is mainly concentrated in two domains: hazard forecasting and risk assessment. Neural networks and ML methods have emerged as the most widely adopted techniques, showing promising performance in tasks such as water inflow prediction, inrush probability evaluation, and water source identification. Notably, from 2022 to 2024, the number of related publications continued to rise, underscoring the growing potential of AI technologies in advancing mine water hazard prevention and control.

As coal mining extends into deeper strata, geological structures and aquifer distributions become increasingly complex, while conventional monitoring methods such as manual inspections and hydrogeological analysis face inherent limitations, including delayed data acquisition, insufficient spatial coverage, and restricted prediction accuracy. These shortcomings are no longer adequate to meet the modern requirements of timeliness, accuracy, and intelligence in water hazard prevention. In recent years, AI has gradually emerged as a powerful tool for mine water hazard prediction, owing to its advantages in large-scale data processing, pattern recognition, and time series forecasting. Methodologically, diverse approaches have been employed: SVM is well suited for classification and regression tasks under small-sample conditions; ensemble learning enhances model robustness and stability through the integration of multiple learners; and DL enables automatic extraction of complex nonlinear features from multi-source sensor data. Supported by these techniques, prediction models have been developed based on historical hydrological records, geological parameters, and online sensor readings, allowing dynamic assessment of water inflow, water level fluctuations, and the probability of inrush events. Compared with traditional empirical approaches, such models significantly improve prediction accuracy and automation, reduce dependence on expert judgment, and provide essential technical support for the development of intelligent mining and unmanned monitoring systems. The distribution of data indicators adopted in existing studies is summarized in Table 6.

As illustrated in Table 6, quantitative indicators for predicting mine water inrush exhibit considerable variability depending on the geological setting and aquifer characteristics. The aquifer thickness typically ranges from a few meters in shallow seams to over 100 m in karstic or fault-controlled aquifers, with thicker layers generally corresponding to stronger hydraulic connectivity and higher water inrush potential. The permeability coefficient of aquifer-bearing strata often varies between 10^−6^ and 10^−2^ m/s, serving as a key determinant of seepage intensity. The specific storage coefficient usually lies within 10^−6^–10^−3^ m^−1^, indicating the unit water volume released per pressure drop in confined aquifers. The aquifer water pressure may range from 0.1 MPa in shallow coal seams to over 3 MPa in deep confined aquifers, significantly influencing water inrush risks. Differences among these benchmark values across studies primarily result from variations in geological formations, measurement techniques (e.g., packer tests vs. tracer methods), and the distinct hydrogeological regimes of different coalfields. Therefore, integrating both quantitative parameter thresholds and geological–structural context provides a more comprehensive assessment of water inrush risks in complex mining environments.

In mine water hazard prediction, rapid and accurate identification of water inrush sources is a critical step. Traditional approaches mainly rely on hydrochemical composition analysis (e.g., concentrations of $Na^{+}$, $Ca^{2+}$, $Cl^{-}$ and ${\mathrm{SO}}_{4}^{2-}$) [110] and expert interpretation. Although these indicators can effectively characterize the hydrochemical differences among various water sources, their discriminative power is significantly reduced in deep mining conditions where multiple aquifers coexist and mixing occurs, due to the overlapping ion signatures. With the advancement of artificial intelligence, researchers have begun to explore intelligent pathways for source identification. Li et al. [111] introduced absorption spectroscopy in combination with a GA-optimized XGBoost model, achieving water source discrimination solely based on spectral data without requiring complex laboratory analysis, which greatly improved efficiency. Similarly, Yan et al. [112] applied laser-induced fluorescence technology and developed an SSA-optimized BPNN for aquifer mixing ratio classification, which achieved higher accuracy, particularly in environments with complex hydrogeological conditions. Overall, AI-based methods have broken through the limitations of conventional approaches and demonstrated unique advantages under multi-source aquifer conditions. Nevertheless, water source identification only represents the preliminary stage in the chain of mine water hazard prevention and control. Advancing towards dynamic prediction of inflow volume and comprehensive risk assessment remains the next critical challenge.

**Figure 12:**
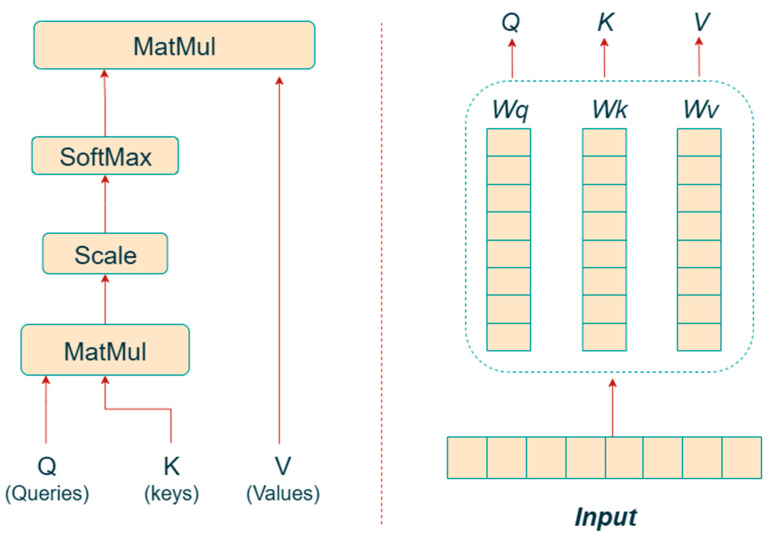
Self-attention architecture diagram. On the left, it shows the computation flow: Queries (*Q*) and Keys (*K*) first undergo a matrix multiplication (*MatMul*), then are scaled, passed through a SoftMax function, and finally multiplied with Values (*V*) via another matrix multiplication to achieve the output. On the right, it depicts how the input is transformed into *Q*, *K*, and *V* using weight matrices $W_{q}$, $W_{k}$ and $W_{v}$, respectively.

Variations in the water content of coal seam roof aquifers directly influence the probability of water inrush events. Traditional prediction methods often fail to capture the complex nonlinear coupling relationships underlying such processes. AI models, with their strong capability for handling nonlinear and non-stationary time series, have increasingly become critical tools for mine water inrush prediction. Yin et al. [35] proposed a hybrid approach combining LSTM networks with the Isolation Forest (IForest) algorithm. By capturing groundwater level dynamics and detecting anomalous fluctuations, this method effectively enhanced the accuracy and timeliness of mine water hazard warnings, enabling early identification of potential inrush risks. To address the limitations of LSTM and GRU models—such as response delays and gradient vanishing—in predicting abrupt changes in water inflow, Yao et al. [113] introduced a self-attention mechanism–based model, with its framework illustrated in Figure 12. This model adaptively adjusts attention weights, emphasizes critical features, and captures sudden trends within hydrological sequences, thereby exhibiting stronger adaptability under complex hydrogeological conditions. Overall, AI-based methods have significantly improved the precision and real-time capability of water inrush forecasting. However, most studies remain constrained by reliance on single-source datasets. The integration of multi-source heterogeneous information into a unified predictive framework has emerged as a key direction for advancing intelligent mine water disaster prevention and control.

Although AI technologies have achieved considerable progress in water source identification and water inflow prediction, the evolution of mine water hazards is often influenced by multiple factors. Relying solely on a single objective is insufficient to comprehensively capture their inherent complexity. Consequently, an increasing number of researchers are shifting towards multimodal prediction frameworks that integrate hydrogeological conditions, mining-induced disturbances, historical water inrush records, and multi-source sensor data [114]. To address the challenges of small sample size and class imbalance, Ye et al. [115] employed the synthetic minority oversampling technique (SMOTE) to expand rare class samples and adopted deep belief networks (DBN) to automatically extract critical features, thereby alleviating the adaptation bottleneck of multimodal frameworks under data-scarce scenarios. Yin et al. [116] combined multiple ML and DL algorithms to detect temporal anomalies in microseismic event data from different perspectives. By incorporating spatiotemporal feature fusion and multi-algorithm collaborative strategies, they realized microseismic data-driven precise early warning of water inrush. Wu’s team [117] tackled the challenge of distinguishing water flow features in underground video streams by proposing a U2Net-based water inrush image recognition model with multi-channel residual attention mechanisms. By simultaneously leveraging RGB and grayscale channel features, their approach effectively suppressed dust and illumination interference, significantly enhancing the model’s robustness in high-noise environments and extending the application boundaries of vision-based modalities in water inrush recognition. Collectively, these studies enrich the methodological system of water inrush prediction and lay the groundwork for the development of unified prediction frameworks based on multi-source data fusion.

In recent years, the rapid development of LLM has provided new approaches and tools for mine water disaster prediction. With their powerful capabilities in cross-modal perception, knowledge reasoning, and multi-task transfer, LLMs hold promise for uncovering latent patterns within complex geological, hydrological, and engineering multi-source datasets. This offers potential pathways to overcome the limitations of conventional single models under small-sample and nonlinear coupling conditions. For example, the “Xiaowu” geological vertical large model, developed by the Xi’an Research Institute of China Coal Technology and Engineering Group, serves as a domain-specific vertical model for geoscience. It integrates geological exploration, hydrogeology, and engineering geology data together with professional knowledge bases, enabling not only the identification of aquifer occurrence conditions but also the prediction of water inrush channels and disaster risk assessment. This demonstrates new application prospects in mine water hazard prediction. Similarly, the AQUAH Agent system developed abroad employs natural language interaction to drive hydrological simulations and disaster prediction. It can automatically retrieve topographic, hydrological, and other multimodal data to complete model configuration, execution, and result interpretation, thus achieving an integrated workflow from prompt input to prediction reporting [118]. These cross-modal intelligent agents highlight the potential of LLM in disaster modeling and automated knowledge reasoning, while also providing new directions for the scalability and intelligence of mine water disaster prediction systems.

Table 7 summarizes the research progress of AI in mine water disaster prediction. It is evident that AI technologies have achieved remarkable advances in water source identification, water inflow prediction, and multi-source data fusion, thereby improving prediction accuracy, timeliness, and spatial coverage. However, current studies still suffer from limitations such as single-dimensional data, restricted model evaluation metrics, and insufficient generalization capability. Future development should focus on the integration of multimodal data, the establishment of intelligent early warning systems, and the incorporation of emerging technologies including large models, digital twins, and transfer learning. These efforts will promote mine water disaster prediction toward full-scenario coverage, systematic modeling, and high reliability, ultimately providing more intelligent prevention and control strategies for mine safety.

### 3.4. Roof Disaster

**Figure 13:**
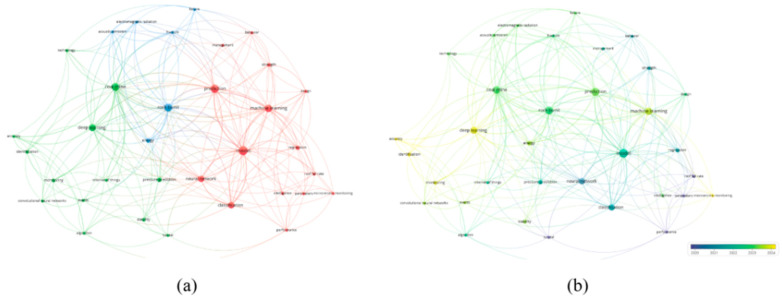
Research focus and temporal evolution of roof disaster with AI. (**a**) Keyword co-occurrence clustering map based on publications from the Web of Science database. (**b**) Temporal distribution of research hotspots.

In coal mining, roof disasters are among the most common and destructive geological hazards, characterized by sudden occurrence, strong concealment, and wide impact. Once triggered, they may not only cause casualties due to roof collapse or impact but also induce secondary disasters such as coal and gas outbursts and water inrush, thereby further increasing the complexity and difficulty of mine safety management. In recent years, as coal mining in China has shifted to greater depths, factors such as high ground stress, intensive fault development, and abnormal mechanical properties of rock strata have significantly increased the difficulty of surrounding rock control, leading to a continuously rising risk of roof disasters [120]. Therefore, achieving accurate monitoring, timely early warning, and intelligent prediction of roof disasters has become an urgent issue in coal mine safety research. According to the keyword co-occurrence map (Figure 13a) and overlay visualization by year (Figure 13b) generated using VOSviewer, current studies mainly focus on two aspects: disaster prediction and the application of modeling methods. Early research predominantly relied on traditional approaches, such as neural networks, for modeling and analysis of roof disasters. In contrast, with the rapid development of AI technologies, DL and ML methods have been widely introduced in recent years and are increasingly integrated with multi-source monitoring data (e.g., microseismic monitoring and acoustic emission) to improve the accuracy and reliability of disaster prediction and identification. Overall, this field is evolving from experience-driven and single-model analysis toward intelligent modeling, multi-source data fusion, and quantitative risk assessment, providing essential technological support for smart mining and intelligent disaster prevention and control.

In recent years, with the deep implementation of the smart mining strategy, AI technologies have been widely applied to the monitoring and prediction of coal mine roof disasters. Leveraging their powerful capabilities in data learning and pattern recognition, AI methods provide novel solutions for roof disaster forecasting. The occurrence of roof disasters is often accompanied by a series of precursor changes, which are continuously collected by underground sensor networks (e.g., stress meters, microseismic monitoring systems, acoustic emission devices, and video sensing equipment). These data, recorded in the form of time series or spatial distributions, reflect the mechanical and energy states of rock masses and constitute the basis for constructing predictive models and early warning mechanisms. However, monitoring data in underground environments are typically nonlinear, non-stationary, and noise-prone, with key precursor signals often obscured by random fluctuations. Thus, how to achieve reliable feature extraction and anomaly detection in complex noisy environments becomes a critical factor in determining whether AI models can truly enhance the sensitivity and stability of prediction. Table 8 further summarizes the commonly used data indicators for roof disaster prediction in coal mines, providing essential support for the development of AI-driven multi-source fusion models.

As shown in Table 8, quantitative indicators related to roof disaster prediction vary according to mining depth, geological conditions, and roof lithology. The vertical stress of the roof in the working face generally ranges from 5 to 30 MPa in most coal mines but may exceed 40 MPa in deep mining zones (>1000 m), where excessive stress accumulation can induce roof collapse or rock bursts. The support working resistance of hydraulic systems typically varies between 3000 and 12,000 kN, depending on the type of hydraulic support and mining height; abnormally high or low resistance readings often indicate potential instability or improper load distribution. Variations in these quantitative benchmarks across different studies mainly arise from geological heterogeneity, distinct rock mechanical properties, and differences in monitoring instrumentation. For instance, in situ stress measurements using hydraulic anchor sensors may yield slightly lower stress values than those derived from numerical inversion models, while differences in lithology (e.g., mudstone versus sandstone roofs) can significantly influence stress distribution and failure modes. Therefore, establishing standardized measurement frameworks and multi-source data fusion approaches is essential to improve the comparability and reliability of roof disaster predictions.

In coal mine roof disaster prediction, mine pressure variation is regarded as the most direct and critical early-warning signal. Traditional mine pressure monitoring relies on devices such as pressure gauges and stress meters to obtain stress evolution trends; however, these methods often exhibit lag effects and insufficient stability in identifying sudden anomalies and revealing periodic patterns. In recent years, AI technologies have been increasingly introduced into mine pressure monitoring to analyze the dynamic processes of stress variation [121,122]. Among various indicators, the working resistance of hydraulic supports serves as the core parameter of mine pressure manifestation, reflecting both periodic and abrupt changes in roof pressure and providing an important basis for roof disaster prediction. Lai et al. [28] employed a hydraulic support pressure monitoring system to construct a PSO-optimized BPNN model, which demonstrated faster convergence and superior fitting of nonlinear periodic patterns compared with traditional approaches. The predictive results accurately captured the periodic fluctuations of mine pressure data, offering valuable guidance for on-site roof management. Chen et al. [123] introduced deep spatiotemporal sequence models (PredRNN, PredRNN++), which significantly enhanced feature extraction and prediction accuracy by capturing spatial correlations among adjacent supports and identifying temporal dynamics through gated units. Overall, AI technologies exhibit distinct advantages in periodic pattern recognition and anomaly detection, effectively compensating for the deficiencies of traditional monitoring methods. Nevertheless, challenges remain in terms of dependence on data quality and the real-time performance of field applications.

In addition to precursor identification based on single monitoring features, recent studies have attempted to construct multi-source data fusion models using AI algorithms to achieve quantitative prediction of roof disasters. Yu et al. [124] incorporated not only rock mechanical parameters and geological conditions into roof deformation prediction but also, for the first time, introduced unsupported time as a key indicator. This improvement rendered the predictions more consistent with actual underground engineering conditions and provided direct guidance for the optimization of support schemes. On the other hand, water infiltration significantly weakens the mechanical properties of roof strata and often serves as a critical trigger in disaster evolution. Addressing this issue, Dong et al. [125] proposed a quantitative prediction method for roof water-yielding capacity based on a CNN optimized by SAA and further integrated electrical, magnetic, and drilling detection techniques to establish a dynamic monitoring and early warning system. This approach achieved deep integration of AI with physical exploration, overcoming the limitations of traditional qualitative assessments. Overall, such studies demonstrate that the introduction of multi-source data and intelligent algorithms enhances the capability to characterize roof disaster evolution and improves prediction accuracy. Nevertheless, challenges remain in ensuring data consistency, strengthening model generalization, and promoting field-scale applications.

The rapid advancement of emerging AI technologies is driving a paradigm shift in coal mine roof disaster prediction, evolving from single-source monitoring toward the deep integration of LLMs, digital twins, and AI agents. In recent years, digital twin approaches have been applied to the modeling and reconstruction of hydraulic support group postures. Utilizing platforms such as Unity3D, these methods enable visualization and dynamic simulation of hydraulic support states, achieving virtual–real mapping of support operation conditions and roof stress distribution. Furthermore, they provide interpretable insights into the mechanisms of roof deformation and collapse risk evolution, offering real-time support for disaster prediction [126]. Meanwhile, domain-specific LLM such as the “Taiyangshi Mine Model” and “Shendong Huizhi” have incorporated key indicators including roof pressure and support status into unified knowledge and data frameworks. By coupling with digital twin platforms, these systems achieve global perception of support anomalies and intelligent early warning of collapse risks. Overall, with the deep convergence of LLMs, digital twins, and AI agents, coal mine roof disaster prediction is advancing from traditional static threshold-based monitoring toward a new stage characterized by virtual-real interaction, real-time evolutionary modeling, and intelligent response. Nonetheless, significant challenges remain in terms of real-time performance and computational cost.

Table 9 summarizes the research progress of AI in roof disaster prediction. The introduction of AI technologies has significantly improved prediction accuracy, timeliness, and spatial coverage. However, existing studies are still constrained by limited data dimensions, single evaluation systems, and insufficient model generalization. Future directions may include multimodal data fusion, cross-modal large models, digital twin platforms, and transfer learning, which can drive end-to-end intelligent modeling across surrounding rock, support systems, equipment, and ground surface. Such approaches are expected to enable full-scenario prediction and risk assessment of roof disasters, thereby enhancing model robustness and practical value under complex geological conditions.

### 3.5. Coal Dust Disaster

**Figure 14:**
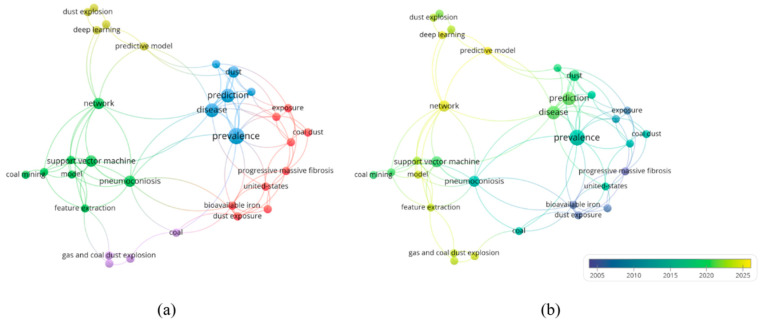
Research focus and temporal evolution of coal dust disaster with AI. (**a**) Keyword co-occurrence clustering map based on publications from the Web of Science database. (**b**) Temporal distribution of research hotspots.

Coal dust disasters represent one of the most prevalent and hazardous types of disasters in coal mine production, characterized by strong concealment, high destructiveness, and wide impact. Their hazards are mainly reflected in two aspects: first, coal dust accumulated underground over time is highly prone to explosion under the action of ignition sources or gas, leading to large-scale casualties and equipment damage; second, long-term inhalation of fine particulate matter in coal dust can easily induce occupational respiratory diseases such as pneumoconiosis, posing serious threats to miners’ health. To systematically review the research progress of AI in the field of coal dust disasters, a thematic search was conducted in the Web of Science Core Collection, and VOSviewer was employed for keyword co-occurrence and temporal evolution analysis (Figure 14a,b). The results indicate that existing research primarily focuses on two directions: coal dust explosion prevention and control, and occupational health risk assessment, with machine learning and deep learning gradually emerging as the core methodologies. Early studies emphasized the identification and diagnosis of health hazards such as pneumoconiosis, whereas in recent years the application of predictive modeling, feature extraction, and optimization methods has been significantly strengthened. This trend suggests that the field is undergoing a transformation from single-focus health risk studies toward intelligent, multidimensional disaster prevention and control strategies.

Effective monitoring and prediction of coal dust disasters rely on the accurate acquisition and analysis of relevant environmental and health data indicators. Although a complete theoretical framework for the mechanism of coal dust explosions has not yet been established, existing studies have demonstrated that key parameters such as dust concentration, temperature, and humidity are closely associated with explosion risk and are therefore widely used as core indicators for risk assessment. With the advancement of sensor and data acquisition technologies, underground coal mines are now capable of real-time collection and dynamic updating of heterogeneous multi-source data, including dust concentration, gas composition, temperature and humidity, airflow velocity and pressure, as well as workers’ health parameters. These developments provide a solid data foundation for AI-based model training and intelligent early warning. Table 10 summarizes the typical parameter indicators commonly used in recent domestic and international studies.

As shown in Table 10, the quantitative ranges of environmental indicators play a decisive role in determining coal dust explosion risk. The coal dust concentration exhibits a critical explosive range between 30 and 300 g/m^3^, depending on particle size, coal rank, and dispersion uniformity. Concentrations below 30 g/m^3^ are generally insufficient to sustain combustion, whereas levels exceeding 300 g/m^3^ tend to suppress flame propagation due to oxygen dilution. Differences in the indicator ranges and conclusions across studies are largely attributed to variations in coal composition, particle size distribution, and laboratory versus in situ experimental conditions. For instance, bituminous coal tends to have lower explosion limits than anthracite due to higher volatile content. Moreover, measurement approaches—such as optical dust sensors versus gravimetric sampling—can yield distinct results due to calibration sensitivity. Hence, harmonizing experimental protocols and defining standardized benchmark ranges are essential for improving the reproducibility and reliability of coal dust explosion prediction and prevention studies.

In the field of coal dust explosion monitoring and prediction, the coupled effects of multiple factors create high complexity and uncertainty in risk identification. Therefore, achieving efficient, accurate, and real-time early warning remains a core challenge for ensuring mine safety. AI technologies, with their advantages in processing heterogeneous multi-source data and extracting complex features, are increasingly demonstrating potential for early monitoring of coal dust explosions. Unlike traditional monitoring approaches that rely on physicochemical parameters such as dust concentration and gas composition, acoustic signal–based recognition methods enable non-contact, real-time, and highly sensitive dynamic monitoring, providing an important complement to early warning systems. Yu et al. [128] transformed transient acoustic signals generated during coal dust explosions into image features and, through wavelet transform, constructed time–frequency coefficient maps that visually revealed differences between explosion and non-explosion acoustic characteristics. Subsequently, to address efficiency bottlenecks in real-time monitoring and large-scale deployment, Yu et al. [129] introduced a lightweight multilayer perceptron model, which significantly improved computational efficiency and resource adaptability while maintaining recognition accuracy. Collectively, these studies highlight that AI technologies have demonstrated high sensitivity and strong application potential in coal dust explosion monitoring and prediction. Nevertheless, further research is needed to enhance multi-source data fusion and adaptability under complex operational conditions.

The hazards of coal dust are not limited to explosion risks but also lie in its long-term health impacts. Prolonged exposure to high concentrations of coal dust can easily induce irreversible damage such as pneumoconiosis. Statistics show that in China, the number of coal miners suffering from pneumoconiosis has exceeded 700,000, with more than 10,000 new cases reported annually, posing a severe threat to workers’ health and imposing a heavy public health and socioeconomic burden [130]. Dust concentration is the core factor determining workers’ exposure risk; however, existing monitoring methods generally suffer from response delays, insufficient spatial coverage, and a lack of intelligent analysis, making it difficult to accurately reflect real working environments in a timely manner. In recent years, AI has provided new approaches for dust monitoring and prediction. Hosseini et al. [131] combined dimensional analysis with neural networks to predict blasting dust concentration. Li et al. [132] applied an LSTM-Attention framework to capture temporal and feature associations of dust dispersion, achieving high-precision modeling and prediction of dust diffusion in open-pit mining scenarios. Chen et al. [133] integrated YOLOv5 with lightweight networks, significantly improving the accuracy and real-time performance of dust detection under image-based monitoring conditions. Collectively, these studies demonstrate the advantages of AI in multi-source data fusion, feature extraction, and dynamic modeling, laying a solid foundation for the intelligent and systematic prevention and control of coal dust disasters.

Table 11 summarizes the research progress of AI in coal dust disaster prediction. AI technologies have achieved significant advances in core tasks such as explosion identification and dust concentration monitoring, demonstrating advantages in high accuracy, rapid response, and flexible deployment, and promoting a transition from experience-driven to data-driven disaster management. However, existing studies still face challenges such as subjective threshold settings, single monitoring perspectives, and insufficient model interpretability and engineering applications. Future research should focus on multimodal data fusion, the development of intelligent early warning systems, and the integration of cutting-edge technologies such as large models and intelligent agents, thereby improving the accuracy, robustness, and scalability of coal dust disaster prediction and providing more systematic intelligent prevention and control strategies for mine safety.

## 4. Summary and Prospects

This study provides a systematic review of AI applications in five major categories of coal mine disasters, namely gas disaster, mine fires, mine water disaster, roof disaster, and coal dust disaster. The findings indicate that methods such as ML, DL, and LLM have achieved continuous progress in feature extraction, risk identification, predictive modeling, and early warning system development. In addition, the integration of multiple sensor networks with simulation platforms has promoted the evolution of disaster prediction toward higher levels of intelligence and systematization. Overall, AI is driving a paradigm shift in coal mine disaster forecasting, moving from experience-based to data-oriented approaches, from single-dimensional analyses to integrated use of heterogeneous information, and from passive response to proactive early warning.

Building upon the detailed analyses and summary tables presented in Section 3, this section further synthesizes the six major challenges that currently constrain the development of AI-driven disaster prediction in coal mines. These challenges are distilled from common issues repeatedly observed across different disaster types, reflecting both shared methodological limitations and domain-specific barriers.

(1) Limited integration across different types of disasters. Existing prediction systems in mine safety are usually developed independently for specific hazards. They lack interoperability of data and coordination among models, which constrains the ability to manage cascading hazard chains. Future research should make use of large-scale foundation models and digital twin mines to establish unified prediction and warning platforms that can provide a dynamic representation of mine-wide risks.
(2) Insufficient fusion of physical mechanisms and data. Current models are mainly driven by data and often lack systematic representation of the mechanisms that generate disasters. Indicator systems and weight assignments continue to rely heavily on expert judgment. A promising pathway is to combine physical mechanism models with artificial intelligence models, supported by physics-informed neural networks and knowledge-augmented foundation models, in order to enhance scientific validity and interpretability.
(3) Data scarcity and limited generalization ability. Coal mine disasters are rare and occur unexpectedly, and most available datasets originate from a single mining area, which limits cross-regional applicability. Future efforts should focus on the construction of shared databases and benchmark datasets covering multiple mining areas. Transfer learning, federated learning, and the generation of synthetic data through virtual simulation can further improve robustness and scalability.
(4) Challenges in engineering deployment and real-time performance. Many studies remain at the laboratory validation stage, while practical applications encounter difficulties such as low accuracy of sensors, limited computing resources, and high maintenance costs. Addressing these issues requires integrated solutions that cover both algorithms and hardware. The combination of edge computing with the industrial internet of things offers a promising direction for enhancing real-time intelligent computation in mining environments.
(5) Lack of autonomous intelligence and collaborative capability. Current systems are mainly designed as decision-support tools, with limited capacity for active learning and dynamic cooperation. Future research should integrate multiple intelligent agents with reinforcement learning to realize adaptive monitoring, assessment, and warning. By combining foundation models with intelligent agents, systems may advance toward proactive recognition, anticipatory intervention, and autonomous evolution.
(6) Bottlenecks in the application of large models. The use of large artificial intelligence models in mining remains in an exploratory phase and lacks established mechanisms for collaboration. Major obstacles include high training and inference costs, limited underground computing power, insufficient domain-specific corpora, inadequate transfer of knowledge, and the risk of producing inaccurate outputs. Future research should promote the development of large models designed for mining applications, integrating geological, sensor, and accident data from multiple modalities. Strengthening causal reasoning and knowledge retention, while combining with agent-based methods, may lead to intelligent decision-making systems capable of planning tasks and simulating risks.

In conclusion, although AI shows substantial potential in the prediction of coal mine disasters, future progress should extend beyond improving the accuracy of individual models. The ultimate goal is to construct unified intelligent systems that can overcome the limitations of single-hazard, single-scale, and single-scenario approaches. By drawing upon foundation models, digital twin technology, and agent-based architectures, the field may achieve a fundamental transformation from experience-based prediction to intelligent autonomy.

## Figures and Tables

**Table 1:** Commonly used machine learning algorithms in coal mine disaster prediction.

Model	Fundamental Principles	Typical Application Scenarios	Advantages	Limitations
Support Vector Machine (SVM)	Constructing the maximum margin hyperplane enables the handling of nonlinear classification problems through kernel functions.	Classification of gas outburst levels [18] and coal temperature prediction [19]	Suitable for small samples and nonlinear problems; highly interpretable.	Noise-sensitive and inefficient with large-scale data.
Decision Tree (DT)	Construct a tree-like decision structure by selecting features for classification based on information gain or Gini index.	Roof collapse risk grading [20]	Highly visual, easy to interpret, and capable of handling missing data effectively.	Prone to overfitting and sensitive to noise in training data.
Random Forest (RF)	An ensemble model of multiple decision trees employs the Bagging strategy to reduce overfitting.	Coal temperature prediction [21] and coal dust explosion prediction [22]	High precision, robust performance, and interpretable feature importance.	The model is relatively complex, resulting in higher training and prediction costs.
AdaBoost	Combine multiple weak classifiers in series, focusing on error samples in each round to enhance overall performance.	Multi-source factor assessment of roof collapse disasters [23]	High accuracy, suitable for complex nonlinear distributions.	Sensitive to noise and demanding in sample quality requirements.
GBDT	Based on the principle of gradient descent, multiple tree models are iteratively trained to minimize the loss function.	Nonlinear regression of gas concentration [24] and mine fire intensity grading prediction [25]	High fitting accuracy, supports feature importance ranking, suitable for complex relationship modeling.	Poor model interpretability, numerous hyperparameters, and lengthy training times.
XGBoost	Enhanced implementation of GBDT employs regularization and second-order derivative optimization to improve generalization and training speed.	Outburst prediction [26]	Fast training speed, regularization prevents overfitting, strong feature selection capability.	Parameters are complex, parameter tuning costs are high, and model structure interpretability is generally poor.
BP Neural Network (BPNN)	The backpropagation algorithm is employed to optimize weights in multilayer perceptrons, making it suitable for nonlinear modeling.	Outburst prediction [27] and mine pressure prediction [28]	Strong nonlinear fitting capability, capable of handling complex variable interactions.	Prone to local optima, reliant on hyperparameter tuning, and lengthy training times.
Convolutional Neural Network (CNN)	Local features are extracted using convolution kernels, commonly employed in modeling images and spatial data.	Water inrush prediction [29] and dust concentration prediction [30]	Automatic feature extraction, suitable for processing images and spatial patterns [31,32].	Limited adaptability to time-series data, requiring substantial computational resources.
Recurrent Neural Network (RNN)	Processing sequential data through a loop structure to preserve contextual information.	Prediction of gas concentration time series [33]	Suitable for time-series modeling, capturing long-term dependencies [34].	Prone to gradient vanishing or exploding, making it difficult to model long sequences.
Long Short-Term Memory (LSTM)	Introducing a gating mechanism based on RNNs alleviates the vanishing gradient problem.	Water inrush prediction [35] and roof disaster prediction [36]	Effectively captures long-term dependencies, suitable for complex time-series tasks [34,37].	High computational complexity, lengthy training time.
Gated Recurrent Unit (GRU)	A simplified recurrent neural network architecture incorporating update and reset gates to control information flow.	Gas concentration prediction [38] and floor water inrush prediction [39]	Structure is relatively simple, training efficiency is high, suitable for medium-to-short-term time series forecasting [37].	The ability to model particularly long temporal dependencies is slightly weaker than that of LSTM.
Generative Adversarial Network (GAN)	Comprising a generator and a discriminator, it enhances the quality of generated data through a game-playing process and can be used for data augmentation.	Sparse Disaster Data Augmentation, Rare Event Simulation, Underground Image Synthesis [30]	Data augmentation capable of generating realistic samples, suitable for few-shot learning tasks.	Training instability, prone to model collapse, and highly sensitive to hyperparameters.

**Table 2:** Common indicators for predicting coal and gas outbursts.

Category	Indicator	Selection Reasons	Measurement Method
Coal Properties	Burial depth D	Greater depth leads to higher ground stress and gas pressure; larger stress makes gas more difficult to release, increasing outburst risk.	Borehole depth measurement
Coal Properties	Coal seam thickness H	Thicker seams indicate larger storage space and higher gas accumulation and pressure, resulting in increased outburst risk.	Coal core thickness measurement
Coal Properties	Coal seam dip angle α	Steeper dips lead to more significant stress concentration; gas is more likely to migrate along bedding planes and accumulate at low positions.	Compass measurement of coal seam dip
Coal Properties	Porosity n	Lower porosity reduces gas release rate; under unfavorable conditions, gas may accumulate and cause outbursts.	Low-field nuclear magnetic resonance (NMR) or mercury intrusion method
Coal Properties	Coal firmness coefficient f	Reflects the coal’s resistance to crushing; smaller values indicate softer coal and higher outburst risk.	Drop hammer or uniaxial compressive strength test
Gas Migration Patterns	Gas content W	Represents the material basis of coal and gas outbursts.	Gas desorption method
Gas Migration Patterns	Gas pressure P	High gas pressure provides the driving force for gas release during outbursts.	Borehole gas pressure measurement
Gas Migration Patterns	Gas emission Qg	Direct indicator of gas outburst magnitude.	Gas emission meter
Gas Migration Patterns	Coal seam gas desorption index $\Delta h_{2}$	Represents gas desorption rate and outburst risk by quantifying gas release per unit time.	Gas desorption velocity method
Stress indicators	Stress concentration coefficient σ	Higher stress concentration promotes coal and gas outburst occurrence.	Borehole stress measurement

**Table 3:** Research progress of AI in coal and gas outburst prediction.

Objective	Algorithm Selection	Indicator Selection	Advantages	Limitations
Outburst prediction	t-SNE + GA + SVM [18]	Outburst accident reports from 2010–2019	Effectively removes redundant information and reduces computational complexity, thereby improving model predictive performance.	Some potential factors are not considered (e.g., human emotions, regional culture); high time cost.
Outburst prediction	PSO + SVM [88]	Gas pressure; initial gas emission rate; burial depth; coal fragmentation type; coal firmness coefficient	Trained with data from multiple mining districts; strong generalization.	Highly dependent on data quality; poor interpretability.
Outburst prediction	XGBoost [26]	Gas content; gas pressure; gas diffusion coefficient; porosity; coal firmness coefficient; initial gas emission rate	Quantifies each indicator’s contribution (high interpretability); maintains high prediction accuracy even with missing features.	Requires many indicator variables; single-source data, weak generalization.
Outburst prediction	GA + SA + BPNN [74]	Gas pressure; gas content; burial depth; coal firmness coefficient; coal fragmentation type; coal-seam thickness	Effectively avoids local optima; shorter prediction time; strong nonlinear fitting ability.	High computational complexity; weak generalization; poor interpretability.
Outburst prediction	RS + GA + BPNN [27]	Gas pressure; gas content; dissolved-gas content; mining depth	RS reduces redundant features; GA optimizes BP weights, improving prediction accuracy and convergence speed.	Not validated on other mine datasets; generalization capability unknown
Outburst risk grading	WOA + ELM [77]	Gas pressure; gas content; initial gas emission rate; coal firmness coefficient; porosity; and 18 types of unsafe behaviors	WOA provides fast global search; compared with conventional ELM, improves prediction accuracy and reduces training time; CBR further manages prediction outputs.	Many indicators and high training cost; performance depends on parameter settings and is sensitive to data quality
Gas concentration prediction	PSO + GA + GRU [38]	Upper-corner gas concentration; working-face gas concentration, etc.	Optimized GRU achieves high accuracy, short training time, and fast iteration; enables gas-concentration warning within 7 s.	Prediction performance highly depends on data; no unified standard for indicator selection, which greatly affects performance.

**Table 4:** Common indicators for predicting coal spontaneous combustion in mines.

Category	Indicator	Selection Reasons	Measurement Method
Gas indicator	CO concentration	Major product of coal low-temperature oxidation in the early stage.	Electrochemical gas sensor; portable multi-gas analyzer
Gas indicator	$O_{2}$ concentration	Continuous consumption due to coal oxidation reaction; decreasing rate is related to combustion reaction rate.	Electrochemical gas sensor
Gas indicator	$CO_{2}$ concentration	Generated in both organic and inorganic decomposition during coal oxidation; concentration changes with reaction progress; especially high in enclosed areas.	Infrared gas analyzer
Gas indicator	$CH_{4}$ concentration	One of the major combustible gases; abnormal increases indicate hidden risks of spontaneous combustion.	Infrared methane analyzer; methane detector
Gas indicator	$C_{2}H_{4}$ concentration	Typical high-temperature pyrolysis product; usually appears when temperature exceeds 110 °C, serving as a marker gas.	Gas chromatography (GC); mass spectrometry (MS)
Gas indicator	$C_{2}H_{6}$ concentration	Appears only at high temperatures; indicates secondary oxidation of methane.	Gas chromatography–mass spectrometry (GC–MS)
Gas indicator	CO$/CO_{2}$ ratio	Key indicator of spontaneous combustion process; higher values indicate intensified oxidation.	
Gas indicator	$C_{2}H_{4}$$/C_{2}H_{2}$ ratio	Used to determine combustion stages; combined with temperature monitoring to predict fire development.	
Temperature indicator	Coal temperature T	Higher coal temperature enhances oxidation; strong correlation with combustion intensity.	Thermocouples; infrared temperature sensors
Temperature indicator	Temperature rise rate dT/dt	Represents coal temperature rise rate per unit time; important indicator for judging spontaneous combustion tendency.	
Environmental indicator	Relative humidity RH	High humidity may inhibit spontaneous combustion but may also increase low-temperature oxidation risk.	Capacitive humidity sensor
Environmental indicator	Airflow velocity v	Influences oxygen supply and heat transfer in goafs and working faces, directly affecting spontaneous combustion risk.	Anemometer; thermal anemometer

**Table 5:** Research progress of AI in mine fire prediction.

Objective	Algorithm Selection	Indicator Selection	Advantages	Limitations
Coal temperature prediction	GA + MK − SVM [96]	$CH_{4}$$\;\mathrm{concentration};\;C_{2}H_{6}$$\;\mathrm{concentration};\;C_{2}H_{4}$$\;\mathrm{concentration};\;CO/CO_{2}$$\;\mathrm{ratio};\;C_{2}H_{4}/C_{2}H_{6}$$\;\mathrm{ratio};\;O_{2}/(CO+CO_{2})$ ratio	High precision, strong generalization capability, and excellent stability	Applicable only to small-sample problems; Relies on specific gas indicators, with data acquisition limitations.
Coal temperature prediction	HGS + RF [21]	$O_{2}$ concentration; CO$\;\mathrm{concentration};\;C_{2}H_{4}$$\;\mathrm{concentration};\;CO/\Delta O_{2}$$\;\mathrm{ratio};\;C_{2}H_{4}/C_{2}H_{6}$ ratio	Can quickly and accurately predict the spontaneous combustion state of working faces; interprets multi-stage oxidation processes; achieves good generalization performance.	Partial data fitting effect still needs improvement; limited applicability to unseen working faces and fire types.
Coal temperature prediction	PSO + GRU [105]	$O_{2}$ concentration; CO$\;\mathrm{concentration};\;CO_{2}$$\;\mathrm{concentration};\;CH_{4}$$\;\mathrm{concentration};\;C_{2}H_{6}$$\;\mathrm{concentration};\;C_{2}H_{4}$ concentration	Maintains stability across the entire temperature range (0–200 °C) with strong generalization capability and robustness; Demonstrates excellent temporal processing capability, suitable for dynamic oxidation processes.	High dependence on input data quality; Weak model interpretability; Limited applicability scenarios.
Coal temperature prediction	BPNN [100]	Activation energy, porosity, moisture content, air flow rate, accumulation time, measurement point location	Reveals the influence of various input indicators on coal spontaneous combustion; Overcomes the limitation of traditional methods that only predict at a single monitoring point.	High dependence on data volume and quality; limited interpretability; restricted applicability scenarios.
Coal spontaneous combustion tendency prediction	SHO + ANN [106]	Moisture (M), Volatile Matter (VM), Fixed Carbon (FC), Ash (A), Total Carbon (C), Hydrogen (H), Nitrogen (N), Oxygen (O), Total Sulfur (S)	High prediction accuracy and strong stability	Data originates from a single coal mine, with unknown generalizability; limited explanatory power.
Mine fire intensity grading prediction	GBDT + LightGBM [25]	$O_{2}$ concentration; CO$\;\mathrm{concentration};\;{Ν}_{2}$ concentration; temperature	As a tree-based model, it can identify key influencing factors through feature importance analysis, offering high interpretability; its metrics are simple and easily obtainable.	Data only from a single mine; not validated in other mines or under different geological conditions; only applicable to gas features; other influencing factors not considered; large deviations may occur in complex fire scenarios.

**Table 6:** Common indicators for predicting mine water disaster.

Category	Indicator	Selection Reasons	Measurement Method
Hydrogeological indicator	Aquifer thickness H	The greater the aquifer thickness, the stronger the water inflow.	Mine hydrogeological observation boreholes; automatic water level meters (floating or pressure type)
Hydrogeological indicator	Permeability coefficient K	Quantitative indicator of rock permeability, positively correlated with water inflow.	Pumping test, packer test, water injection test
Hydrogeological indicator	Specific storage coefficient Ss	Reflects the volume of water released per unit aquifer volume per unit decline in hydraulic head.	Laboratory consolidation test; numerical simulation analysis
Hydrogeological indicator	Aquifer water pressure P	Static water pressure at the aquifer bottom; greater pressure indicates stronger water inrush risk.	Pressure sensors
Hydrogeological indicator	$\mathrm{Hydrochemical}\;\mathrm{ion}\;\mathrm{concentration}\;(e.g.,\;{\mathrm{SO}}_{4}^{2-}$$,\;Cl^{-}$)	Ion concentration characteristics in water, used to identify aquifer sources.	Ion chromatography; titration
Geological structure indicator	Fault density	Regional fault development degree determines the potential water inrush pathways.	Borehole three-dimensional seismic exploration
Geological structure indicator	Rock mass fracture density Dc	Number of rock fractures per unit length, reflecting the possibility of water conduction through dissolution channels.	CT scanning; borehole television
Mine drainage indicator	Drainage volume Q	Increase in drainage volume reflects the risk of water inrush.	Mine drainage flow meters

**Table 7:** Research progress of AI in mine water disaster prediction.

Objective	Algorithm Selection	Indicator Selection	Advantages	Limitations
Water inrush source identification	GWO + SVM [110]	$\mathrm{Concentrations}\;\mathrm{of}\;Na^{+}$ $,\;Ca^{2+}$ $,\;Mg^{2+}$ $,\;Cl^{-}$ $,\;SO_{4}^{2-}$ $,\;HCO_{3}^{-}$	Improves identification accuracy by eliminating mixed water samples; reduces misjudgment in aquifer source identification; good operational stability and generalization.	Model performance depends on sample size and quality; limited application scope; does not consider multi-source water mixing identification.
Water inrush prediction	WOA + CNN + SVM [29]	Aquifer thickness, permeability coefficient, fault dip angle	Performs well in predicting water inrush risk during borehole drilling; retains high stability.	Indicator selection limited; only considers aquifer parameters and fault dip angle; lacks validation under complex multi-factor conditions.
Water inrush prediction	LSTM + IForest [35]	Borehole water level historical data	Overcoming the limitation of traditional methods that can only handle a single water source; IForest is highly sensitive to anomalies, addressing the failure of traditional models under extreme conditions.	High data dependency; if borehole distribution is unreasonable or correlation is low, critical water source information may be overlooked.
Floor water inrush prediction	GRU [39]	Coal seam inclination, coal seam thickness, fault dip angle, mining depth, aquifer pressure	Strong temporal dynamic capture capability; high prediction accuracy.	Highly data-dependent; does not address complex scenarios involving mixed water ingress from multiple aquifers, resulting in limited generalizability.
Floor water inrush prediction	Transformer [119]	Borehole water level time series, rainfall, aquifer pressure	Leveraging transfer learning effectively mitigates data sparsity issues and delivers strong generalization capabilities; excels at capturing multivariate and long-time-series patterns.	The effectiveness of transfer learning is highly dependent on the consistency of geological conditions between the two mining areas. The model employs zero-shot prediction and does not account for the impact of real-time mining disturbances on water levels in the target mining area.

**Table 8:** Common indicators for predicting roof disaster.

Category	Indicator	Selection Reasons	Measurement Method
Stress indicator	$\mathrm{Vertical}\;\mathrm{stress}\;\mathrm{of}\;\mathrm{roof}\;\mathrm{in}\;\mathrm{working}\;\mathrm{face}\;\sigma_{z}$	Magnitude of vertical stress borne by the roof, excessive stress may induce roof collapse.	Stress meters (hydraulic anchor sensors), microseismic monitoring system
Stress indicator	$\mathrm{Support}\;\mathrm{working}\;\mathrm{resistance}\;F_{s}$	Abnormal variations in the support force provided by hydraulic supports may indicate roof subsidence.	Hydraulic support monitoring and control system
Stress indicator	$\mathrm{Lateral}\;\mathrm{stress}\;\mathrm{of}\;\mathrm{roof}\;\mathrm{rock}\;\sigma_{x}$	The horizontal stress distribution within the rock mass is closely related to the stability of the roof.	Rock stress meters, in situ stress measurement system
Stress indicator	Roof subsidence $\delta_{z}$	The vertical subsidence distance of the roof strata during mining operations is a critical parameter for identifying precursors to roof falls.	Multipoint extensometers (MPBX), laser displacement sensors, optical fiber monitoring system
Geological indicator	$\mathrm{Roof}\;\mathrm{thickness}\;H_{t}$	Influences bearing capacity and overall stability of the roof.	Geological mapping, core logging, geological drilling
Geological indicator	$\mathrm{Distance}\;\mathrm{to}\;\mathrm{fault}\;\mathrm{or}\;\mathrm{weak}\;\mathrm{strata}\;d_{f}$	Shorter distance to faults or weak strata increases stress concentration and risk of roof failure.	3D geological modeling, seismic exploration, or borehole television
Mining activity indicator	$\mathrm{Advance}\;\mathrm{rate}\;v_{m}$	The advance rate per unit time at the working face; rapid progress causes a lag in support response.	Field records, coal cutting machine parameters
Mining activity indicator	$\mathrm{Mining}\;\mathrm{depth}\;H_{m}$	The vertical depth of the mining location relative to the ground surface indicates that stress is more concentrated at greater depths.	Geological survey data
Environmental indicator	Humidity/seepage conditions W	Affects rock mass strength and roof support stability.	Hydrological monitoring, borehole seepage sensors
Stress indicator	$\mathrm{Vertical}\;\mathrm{stress}\;\mathrm{of}\;\mathrm{roof}\;\mathrm{in}\;\mathrm{working}\;\mathrm{face}\;\sigma_{z}$	The roof is subjected to vertical stress; excessive stress may cause roof collapse.	Stress meters (hydraulic anchor sensors), microseismic monitoring system
Stress indicator	$\mathrm{Support}\;\mathrm{working}\;\mathrm{resistance}\;F_{s}$	Abnormal variations in the support force provided by hydraulic supports may indicate roof subsidence.	Hydraulic support monitoring and control system
Stress indicator	$\mathrm{Lateral}\;\mathrm{stress}\;\mathrm{of}\;\mathrm{roof}\;\mathrm{rock}\;\sigma_{x}$	The horizontal stress distribution within the rock mass is closely related to the stability of the roof.	Rock stress meters, in situ stress measurement system

**Table 9:** Research progress of AI in roof disaster prediction.

Objective	Algorithm Selection	Indicator Selection	Advantages	Limitations
Mine pressure prediction	PSO + BPNN [28]	Hydraulic support working resistance data	Fast convergence; high prediction accuracy	Trained solely on data from a single coal mine working face, its generalization capability remains to be verified.
Mine pressure prediction	SVM [122]	Roof subsidence, advance rate, working face length, coal seam thickness	Incorporates physical constraints; high interpretability; strong processing of multidimensional data	Limited generalization capability; no comparison with state-of-the-art models. Its competitiveness among current leading approaches cannot be verified.
Roof disaster prediction	LSTM [36]	Pillar stress, roof beam angle, advance rate, coal seam thickness, etc.	For the first time, the attitude and load parameters of hydraulic supports are integrated to comprehensively reflect the coupled state between the supports and the surrounding rock.	Requires extensive historical load data; does not account for multi-source interference.
Roof fall prediction	GA + Fuzzy Inference System (FIS) [127]	Mining height, cover depth, support parameters, etc.	Strong uncertainty handling capabilities; high interpretability.	Fewer factors considered, with key elements such as groundwater and geological structures omitted; High dependence on specific datasets.

**Table 10:** Common indicators for predicting coal dust explosion.

Category	Indicator	Selection Reasons	Measurement Method
Environmental indicator	$\mathrm{Coal}\;\mathrm{dust}\;\mathrm{concentration}\;C_{\mathrm{dust}}$	The mass of coal dust per unit volume of air is closely related to its explosion hazard.	Optical dust sensors, light-scattering analyzers
Environmental indicator	$\mathrm{Oxygen}\;\mathrm{concentration}\;C_{O_{2}}$	Oxygen content affects the composition of combustible mixtures.	Gas concentration analyzers, portable gas detectors
Environmental indicator	Temperature T	The tendency for coal dust explosions increases with rising temperatures.	Temperature sensors
Environmental indicator	Humidity H	High relative humidity can reduce suspension stability and affect coal dust ignition.	Humidity sensors
Environmental indicator	$\mathrm{Airflow}\;\mathrm{velocity}\;V_{air}$	High airflow may cause dust resuspension and increase the potential for dust dispersion.	Anemometers, ultrasonic wind speed sensors
Coal dust physical property	$\mathrm{Median}\;\mathrm{particle}\;\mathrm{size}\;D_{50}$	Indicates that 50% of particles are smaller than this median particle size, affecting suspension stability and flammability.	Laser particle size analyzers
Coal dust physical property	$\mathrm{Volatile}\;\mathrm{fraction}\;V_{f}$	The higher the content of combustible components, the greater the explosive sensitivity.	Industrial analysis (drying, ash, volatile determination)
Comprehensive indicator	$\mathrm{Explosion}\;\mathrm{index}\;K_{st}$	Characterizes the intensity and severity of explosions.	Explosion tests (20 L spherical vessel)

**Table 11:** Research progress of AI in coal dust disaster prediction.

Objective	Algorithm Selection	Indicator Selection	Advantages	Limitations
Coal dust explosion prediction	SVM [134]	Volatile content, humidity, combustion duration, coal dust concentration	High prediction accuracy enables precise classification between explosive and non-explosive states.	Indicators lack comprehensive coverage; parameters exhibit high sensitivity.
Coal dust explosion prediction	RF [22]	Particle size, dust concentration, calorific value	Stable training even with small samples; Combined with SHAP method to reveal the influence weights of each metric, offering high interpretability.	Limited applicability in high-concentration scenarios; Input metrics lack comprehensive coverage.
Dust concentration prediction	LSTM + Attention [132]	Temperature, relative humidity, airflow velocity, wind pressure	Strong ability to capture complex relationships; enhanced resistance to interference from input fluctuations.	Limited capability in predicting extreme scenarios; Relies on data integrity.
Dust concentration prediction	WGAN + CNN [30]	Dust concentration, wind speed, temperature, methane concentration	Effectively addressing the small-sample problem, WGAN provides ample high-quality data for subsequent predictions; it demonstrates excellent adaptability across different working faces and is suitable for diverse underground scenarios.	High computational costs; dependent on the quality of raw data

## Data Availability

The raw data supporting the conclusions of this article will be made available by the authors on request.
